# OCNDS core features are conserved across variants, with loop-region mutations driving greater symptom burden

**DOI:** 10.3389/fnhum.2025.1589897

**Published:** 2025-07-03

**Authors:** Elena D. Bagatelas, Maahin Manzoor Khan, Gabrielle V. Rushing

**Affiliations:** ^1^Department of Neuroscience, Vanderbilt Brain Institute, Vanderbilt University, Nashville, TN, United States; ^2^CSNK2A1 Foundation, San Francisco, CA, United States

**Keywords:** OCNDS, CSNK2A1, CK2, Okur-Chung, missense, genotype–phenotype, Simons searchlight

## Abstract

**Introduction:**

Okur-Chung Neurodevelopmental Syndrome (OCNDS) is an ultra-rare genetic disorder caused by *de novo* mutations in the *CSNK2A1* gene, which encodes the catalytic subunit of protein kinase CK2α. OCNDS is characterized by global developmental delay, intellectual disability, speech and language deficits, and other multi-system symptoms. Although prior reports have described considerable phenotypic variability, the relationship between specific CK2α variant locations and symptom presentation remains poorly defined.

**Methods:**

We analyzed natural history data from 48 individuals with pathogenic or likely pathogenic *CSNK2A1* missense variants enrolled in Simons Searchlight. Variants were categorized by their location in conserved CK2α protein domains, specifically distinguishing between loop (e.g., glycine-rich loop, p+1 loop) and non-loop regions. We evaluated symptom burden across organ systems, age at diagnosis, and adaptive functioning using caregiver-reported surveys.

**Results:**

All individuals reported speech/language delay, with additional common features including global developmental delay, neurological symptoms, and gastrointestinal issues. Variants in loop regions were associated with significantly younger age at diagnosis and a higher frequency of hypotonia. Mutations in the glycine-rich loop—known to bind both ATP and the regulatory CK2β subunit—were linked to significantly higher symptom burden and more non-seizure neurological symptoms. No significant differences were observed between variant locations for core features such as sleep issues, intellectual disability, or speech delay..

**Discussion:**

Our findings suggest a core OCNDS symptom profile that is conserved across genotypes, with loop-region variants contributing to increased symptom burden. These results reinforce the relevance of conserved functional domains in driving phenotype severity and support the use of mutation location as a potential biomarker to stratify patients for therapeutic prioritization. Further studies incorporating functional assays and larger cohorts are needed to elucidate mechanisms of variant-specific pathogenesis and guide personalized intervention strategies.

## Introduction

Recent literature has shed light on evolved diagnostic tools and the diverse range of neurodevelopmental disorders resulting from *de novo* variants in genes (Brunet et al., 2021; López-Rivera et al., 2020). Since phenotypes across neurodevelopmental disorders are heterogeneous and can range in severity, whole exome/genome sequencing has facilitated diagnosis and subsequent discovery of genotype–phenotype relationships for genetic disorders (Savatt and Myers, 2021; van der Sanden et al., 2023) including the identification of Okur-Chung Neurodevelopmental Syndrome (OCNDS), an ultra-rare genetic disorder caused by heterozygous mutations in the *CSNK2A1* gene encoding protein casein kinase 2 alpha subunit 1 (Okur et al., 2016). OCNDS is typically caused by *de novo* mutations in *CSNK2A1*, with some autosomal dominant inherited cases noted in literature (Belnap et al., 2023; Goel and O’Donnell, 2024; Ramadesikan et al., 2024). Typical clinical characteristics associated with OCNDS are global developmental delay, dysmorphic facial features, hypotonia, intellectual disabilities, and difficulty feeding (Chung and Okur, 2022). There are also broadly reported nonspecific clinical features reported in the literature, such as behavioral problems, disrupted sleep patterns, seizures, and abnormal growth (i.e., short stature) (Jafari Khamirani et al., 2022; Xu et al., 2020). To date, there are no direct therapeutic treatments for OCNDS, however the most common treatments focus on symptom management. Thus, there is an urgent need to better understand genotype–phenotype correlations and how they may translate to improved treatments for patients with OCNDS.

Casein kinase 2 (CK2) is a serine/threonine kinase that phosphorylates proteins involved in processes such as cell viability, circadian rhythm, cell cycle control and proliferation among other functions (Borgo et al., 2021; Ghani et al., 2024; Poole et al., 2005). CK2 is characterized by several distinct features that differentiate it from other serine/threonine protein kinases including the ability to use both GTP and ATP as phosphate donors, high basal activity, the catalytic subunit containing a large number of conserved basic residues, and ability to recognize phosphoacceptor sites specified by acidic residues (Sarno et al., 1999). It is a tetramer comprised of four subunits, with the alpha subunit containing the regions for catalytic activity and the *β* subunit serving a regulatory role; the CK2 holoenzyme is comprised of a α2β2 stoichiometry (Niefind et al., 2007). Prior reviews highlight that the majority of CK2α variants observed in OCNDS are missense mutations that cluster in highly conserved functional domains and key structural regions, including the Glycine (Gly)-rich loop, activation loop, and p + 1 loop (Unni et al., 2022). Gly-rich loops are a common functional domain of protein kinases and they are one of the most critical structures participating in nucleotide binding, substrate recognition, enzyme catalysis, and overall regulation of activity (Bossemeyer, 1994). Notably, ~23% of individuals with missense variants in our cohort were in the Gly-rich loop. The Lys198Arg (K198R) mutation in the p + 1 loop is considered a mutation hotspot as it is the most frequently observed variant, occurring in approximately 33% of OCNDS patients (Akahira-Azuma et al., 2018; Chiu et al., 2018; Nakashima et al., 2019; Okur et al., 2016; Owen et al., 2018; Wu et al., 2021). There are 17 individuals with this variant in the Searchlight cohort representing ~35% of the missense variants. Lys198 is described as the key residue that determines the anion binding potential of the p + 1 loop (Sarno et al., 1996, 1997); as such, it is not surprising that an arginine substitution would disrupt CK2α activity. Furthermore, existing phenotyping studies suggest that OCNDS patients with variants in the Gly-rich loop are associated with a more diverse and heterogeneous clinical presentation (Wu et al., 2021). Since these key structural regions are highly conserved across kinases, we aimed to explore if regional mutations have varying effects on OCNDS phenotypes.

It is well illustrated that a wide range of clinical features are associated with mutations in the *CSNK2A1* gene (Akahira-Azuma et al., 2018; Chiu et al., 2018; Nakashima et al., 2019; Okur et al., 2016; Owen et al., 2018; Wu et al., 2021). However, it is not well understood how mutations in specific domains of the CK2α protein correlate with the phenotypic spectrum of OCNDS. Here we present analyses of Simons Searchlight natural history data to assess genotype–phenotype correlations in patients with OCNDS. Simons Searchlight,^1^ funded by the Simons Foundation Autism Research Initiative (SFARI), is an international, online research program that builds natural history databases for rare genetic conditions associated with neurodevelopmental disorders. Speech delay/disorders were present in all OCNDS patients in our cohort. We observed that the majority of OCNDS patients in our cohort consistently score below average for the typical developmental trajectory for multiple domains assessed by the Vineland-III regardless of variant location. Additionally, the majority of OCNDS patients with sleep disturbances met the clinical cutoff for pediatric sleep disorders. Furthermore, we compared individuals with OCNDS that have mutations within conserved loop regions of CK2α to those with mutations in other (non-loop) regions to assess how mutations in different domains correlate with patient symptoms. We highlight that individuals with mutations in conserved loop regions of CK2α are diagnosed at a younger age than individuals with mutations in other protein segments and are more likely to present with low muscle tone as a symptom. We also compared individuals with mutations in CK2α residues that interact with CK2β (i.e., all patients with variants in the Gly-rich loop in our cohort) to individuals with mutations in residues that do not bind CK2β. Our goal was to assess if the interaction with the regulatory CK2β subunit influenced symptom presentation. We found that individuals with mutations in the Gly-rich loop present with a higher symptom burden, report a higher number of neurological (non-seizure) symptoms, and exhibit a trend of earlier OCNDS diagnosis age. We hope by further refining the phenotypic spectrum and increasing awareness of OCNDS that clinicians will more readily consider genetic testing when symptoms are first observed.

## Results

### Participants

**Figure 1:**
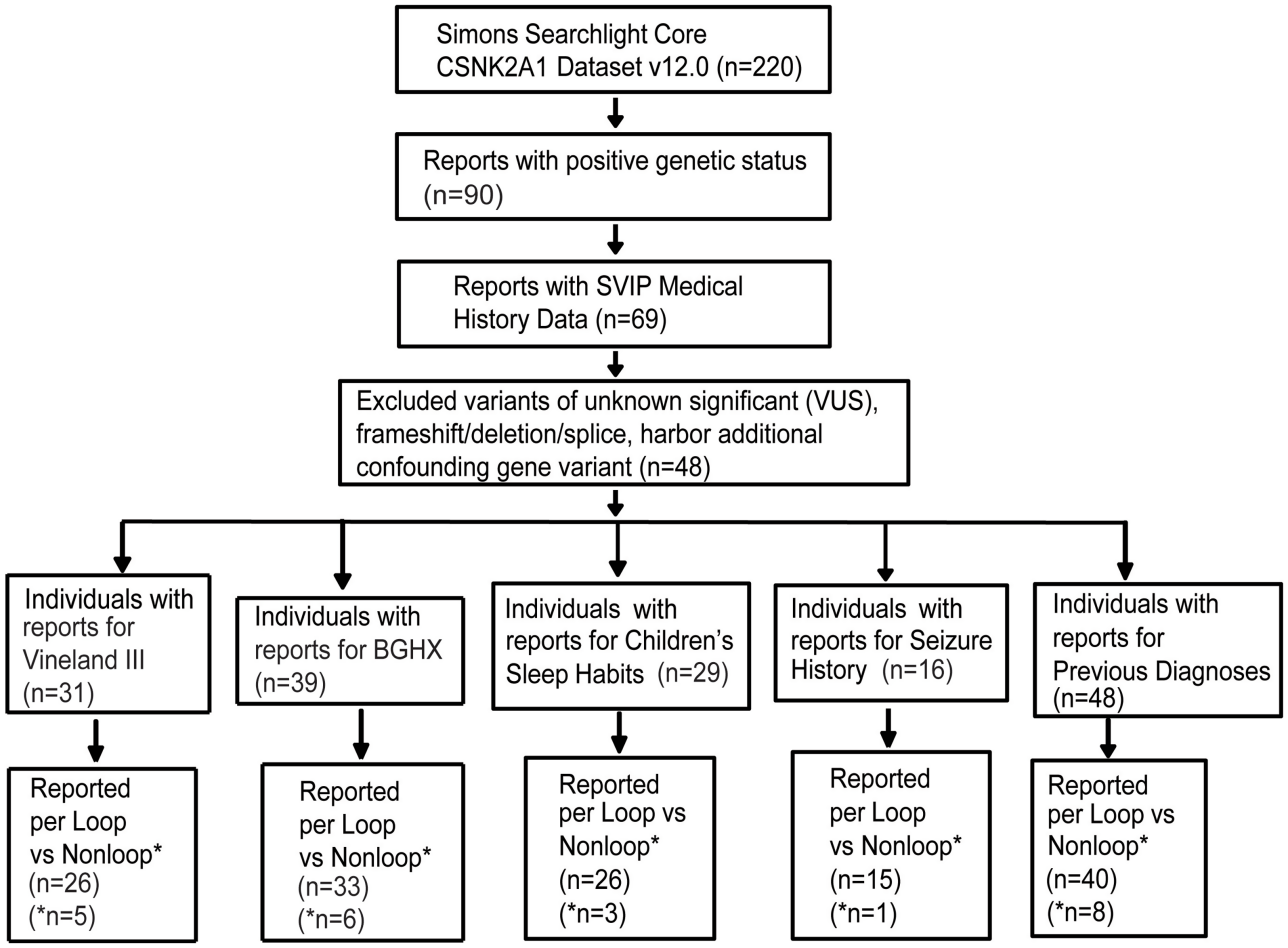
Missense variants included in analysis filtered according to the steps listed in the flow chart. Completed survey responses vary in number. BGHX, Background history questionnaire; SVIP, Simons variation in individuals project.

At the time of data export (May 2024), the *CSNK2A1* dataset held information on 220 individuals including individuals with OCNDS and unaffected family members. Out of 220, 90 individuals had a confirmed genetic report. Since the majority of reported OCNDS cases are missense mutations (Ballardin et al., 2022) and different disease mechanisms have been suggested based on variant type (Caefer et al., 2022; Dominguez et al., 2021a), we narrowed our analyses to include only individuals with missense variants classified as pathogenic or likely pathogenic. We excluded 6 variants of uncertain significance (VUS), 2 deletions, 1 splice variant, 1 frameshift variant, 1 nonsense variant, 1 duplication, and 10 individuals with other reported genetic variants (confounds) that could contribute to symptom presentation. Figure 1 is a flow chart indicating participant counts for each dataset analyzed; excluded participants are summarized in Supplementary Table S1.

**Figure 2:**
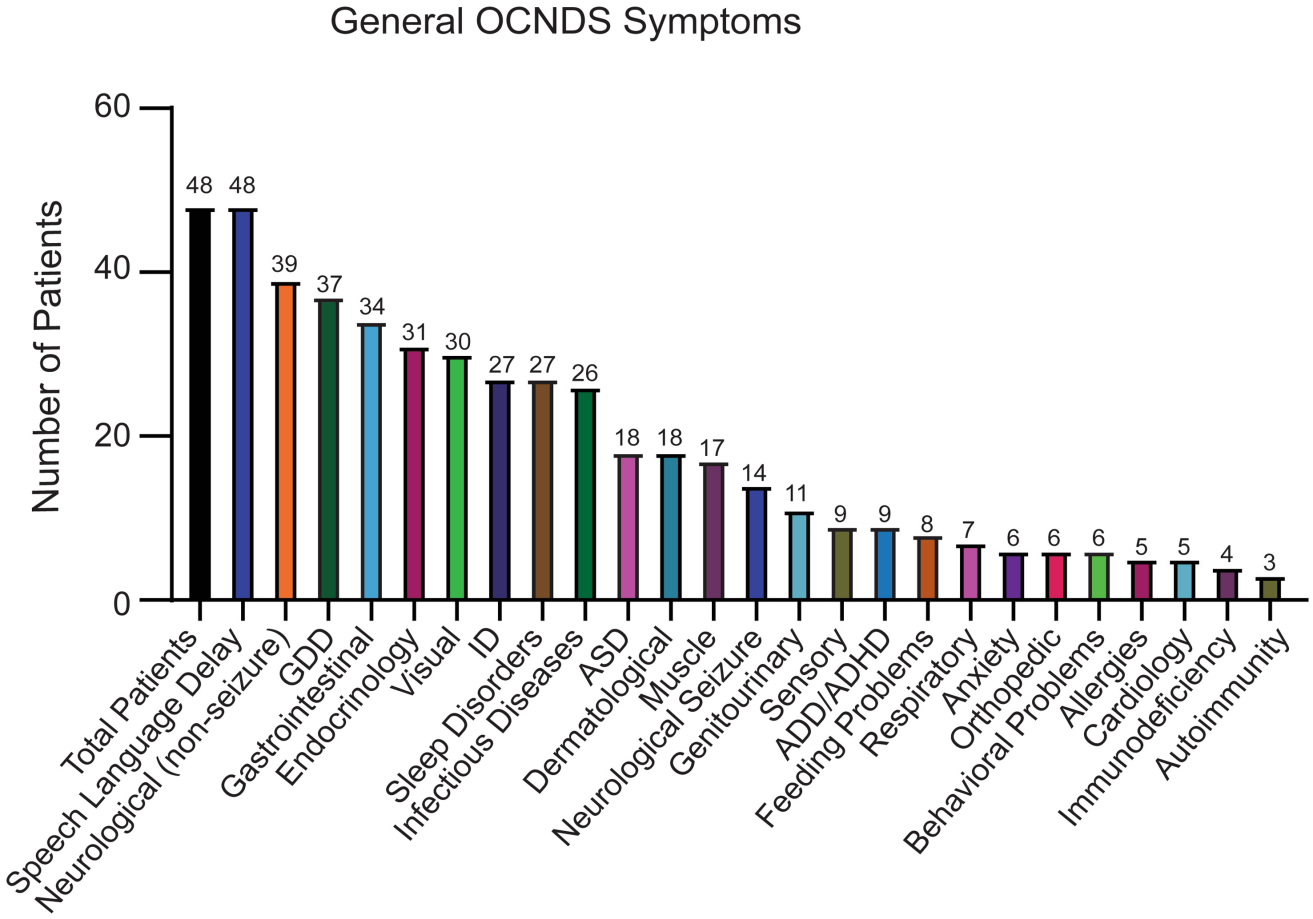
Symptoms reported in OCNDS patients harboring missense variants. Data obtained from the SVIP, Simons variation in individuals project medical history interview survey and previous diagnoses summary.

Out of the remaining 69 confirmed reports, only 48 individuals had specific variant annotations and a completed medical history summary. Most patients in the dataset harbored *de novo* missense mutations except for one case of familial inheritance. The baseline ages of participants ranged from 1 to 31 years, with a mean age of 7 years and 5 months. Out of the 48 patients with missense variants, the top reported symptoms were speech/language disorder/delay (100%), neurological (non-seizure, 81%), global developmental delay (77%), and gastrointestinal (71%) (Figure 2).

### Expanding genotype–phenotype associations

**Figure 3:**
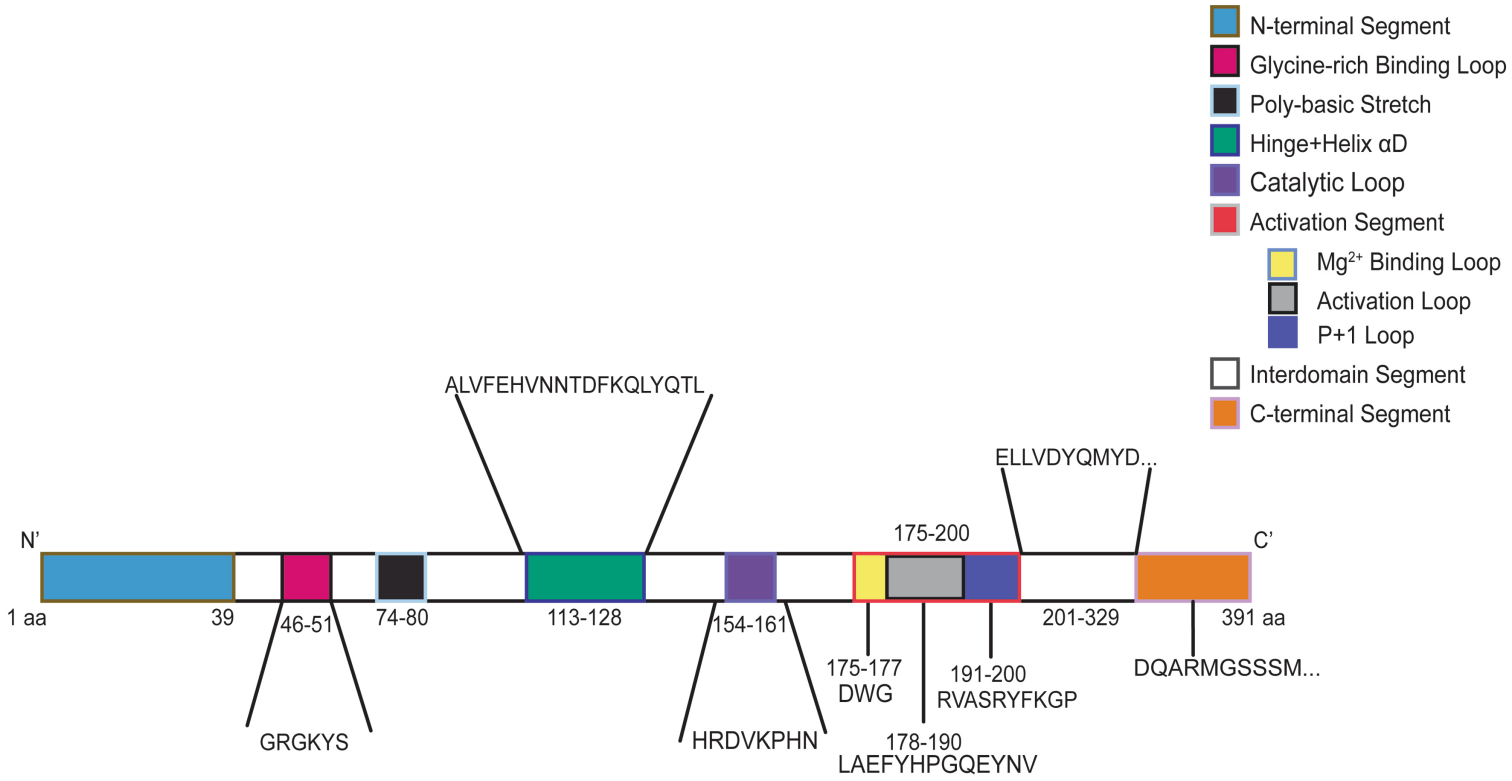
Graphic representation of CK2α protein. Numbers indicate amino acid residues and colors correspond to labeled protein segments.

*CSNK2A1* is made up of 391 amino acids and is comprised of 10 domain segments: N-terminal, Gly-rich binding loop, poly-basic stretch, hinge+ helix αD, catalytic loop, activation segment (comprised of Mg^2+^ binding loop, activation loop, and p + 1 loop respectively), interdomain segments, and C-terminal (Borgo et al., 2021; Sarno et al., 1997, 1999) (Figure 3). The interdomain segments represent the intervals of amino acids that are otherwise unclassified. We classified OCNDS patient mutations according to location within the different domains and calculated the ratio of mutations to number of amino acids (Table 1). We observed missense mutations in every segment except the hinge + helixαD and C-terminal segments, with the p + 1 loop having the highest number of patients (ratio of observed patients/amino acids: 2.1) followed by the Gly-rich loop (ratio 1.83) consistent with previously published work on mutation clusters (Chiu et al., 2018; Unni et al., 2022). The lowest observed ratios were the interdomain and N-terminal segments (0.02 and 0.10, respectively). Notably in our dataset, >60% of patients with a mutation in the activation segment have the same missense mutation (K198R).

**Table 1:** Number of patients per CK2a protein segment.

CK2a protein segment	Amino acid range	Total # of amino acids	Number of patients	# Patients/# amino acids (observed percentage)
N-terminal segment	1–39 aa	39	4	0.10
Gly-rich binding loop (ATP/GTP Binding)	45–53 aa	6	11	1.83
Polybasic stretch	74–88 aa	7	1	0.14
Hinge + Helix αD	113–128 aa	16	0	0.00
Catalytic loop	154–161 aa	8	2	0.25
Activation segment	175–200 aa	26	27	1.03
Mg^2+^ binding loop	175–177 aa	3	3	1.00
Activation loop	178–190 aa	13	3	0.23
P + 1 loop	191–200 aa	10	21	2.10
Interdomain segment	201–330 aa	130	3	0.02
C-terminal segment	331–391 aa	61	0	0.00

Number of patients with variants reported in each protein segment. The table includes the range of amino acids per segment, total number of amino acids per segment, number of patients with missense mutations per segment within our cohort and observed percentage of patient mutations per segment.

**Figure 4:**
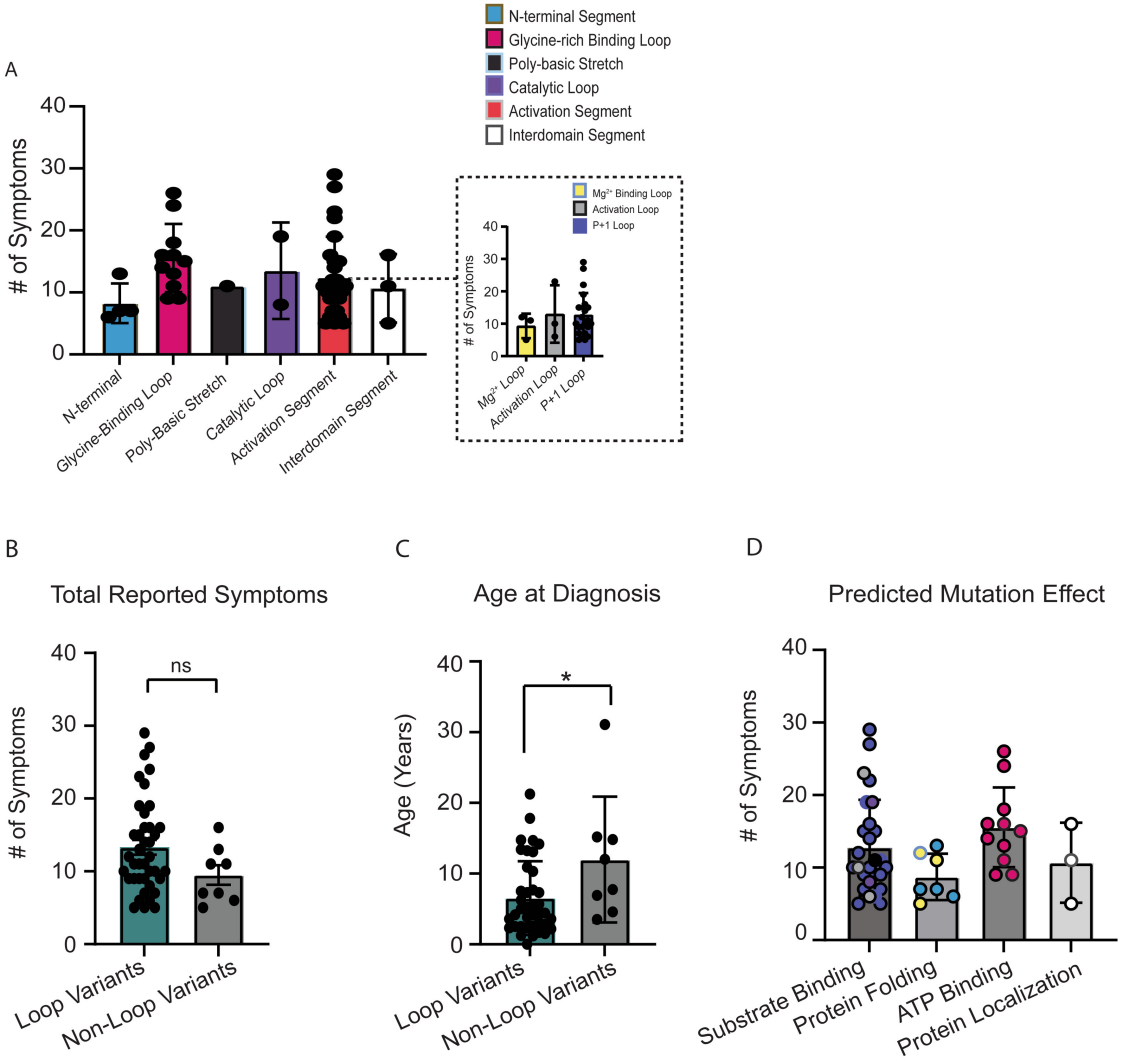
**(A)** Number of OCNDS symptoms reported by patients in each protein segment. Differences in total number of reported symptoms were not statistically significantly different across segments (Kruskal-Wallis test, *p* = 0.28). Inset shows distinct loops within the activation segment. **(B)** Individuals with missense variants residing in a loop segment of the protein (i.e., *glycine-rich loop, catalytic loop, Mg^2+^ −binding loop, activation loop, and p + 1 loop*) reported a higher number of symptoms than individuals with missense variants in other segments (i.e., *N-terminal, polybasic stretch, and interdomain*) but difference was not statistically significant (Mann–Whitney test, *p* = 0.14). **(C)** We had a recorded age at genetic diagnosis for 47/48 (98%) of individuals with missense variants. Individuals with loop variants (*n* = 39) in *CSNK2A1* are diagnosed at a younger age compared to individuals with non-loop variants (*n* = 8) (Mann–Whitney test, *p* = 0.03). **(D)** Number of OCNDS symptoms reported by patients compared by predicted/known protein effect based on Unni et al. (2022); difference was not significant (Kruskal-Wallis test, *p* = 0.09). *p-value is less than 0.05.

We first attempted to compare total OCNDS symptom burden across all protein segments (Figure 4A). Differences were not significant (*p* = 0.09) likely due to the large distribution of sample sizes across segments and our limited total sample size. We observed that segments designated as having loop structures had a higher number of patients and a higher number of reported symptoms per patient. Since loops are highly conserved regions that are important for CK2α function, we further segregated the variants into loop (Gly-rich binding loop, catalytic loop, Mg^2+^ −binding loop, activation loop, p + 1 loop) or non-loop (N-terminal, poly-basic stretch, hinge+helixαD, interdomain, and C-terminal) groups for further comparisons. When we compared the total number of reported symptoms between groups, we observed that individuals with variants in loop regions reported a higher number of symptoms as compared to non-loop regions but this difference was not statistically significant (*p* = 0.14) (Figure 4B); individual-level symptoms by category are reported in Supplementary Table S2. We then compared if age at OCNDS diagnosis differed between loop and non-loop variants. We had a recorded age at genetic diagnosis for 47/48 (98%) patients (*n* = 39 loop, *n* = 8 non-loop). We observed that individuals with loop variants in *CSNK2A1* were diagnosed at a significantly younger age than individuals with non-loop variants (*p* = 0.03) (Figure 4C). For a subset of individuals with non-missense variants, we had medical history data available and compared total number of symptoms to our missense cohort. We observed that for symptom count, most non-missense individuals resembled non-loop individuals (Supplementary Figure S1).

To explore potential functional differences between loop and non-loop variants, we classified variants based on predicted or reported biochemical consequences using data from Unni et al. (2022). Functional categories included altered substrate binding/specificity, altered ATP binding, protein folding, and subcellular localization. We found that loop-region variants were predominantly associated with disrupted substrate or ATP binding, while non-loop variants more often affected protein folding or localization (annotated in Supplementary Table S2). Individuals with variants predicted to alter substrate or ATP binding exhibited a non-significant trend of higher total symptom count compared to those with variants affecting folding or localization (Figure 4D) (*p* = 0.09). These results suggest a potential link between specific functional disruptions and overall symptom burden, with differences in functional consequences between loop and non-loop variants. Given these observed functional distinctions, we proceeded to assess whether loop and non-loop variants were associated with differing patterns of symptom prevalence.

### Neurological symptoms

**Figure 5:**
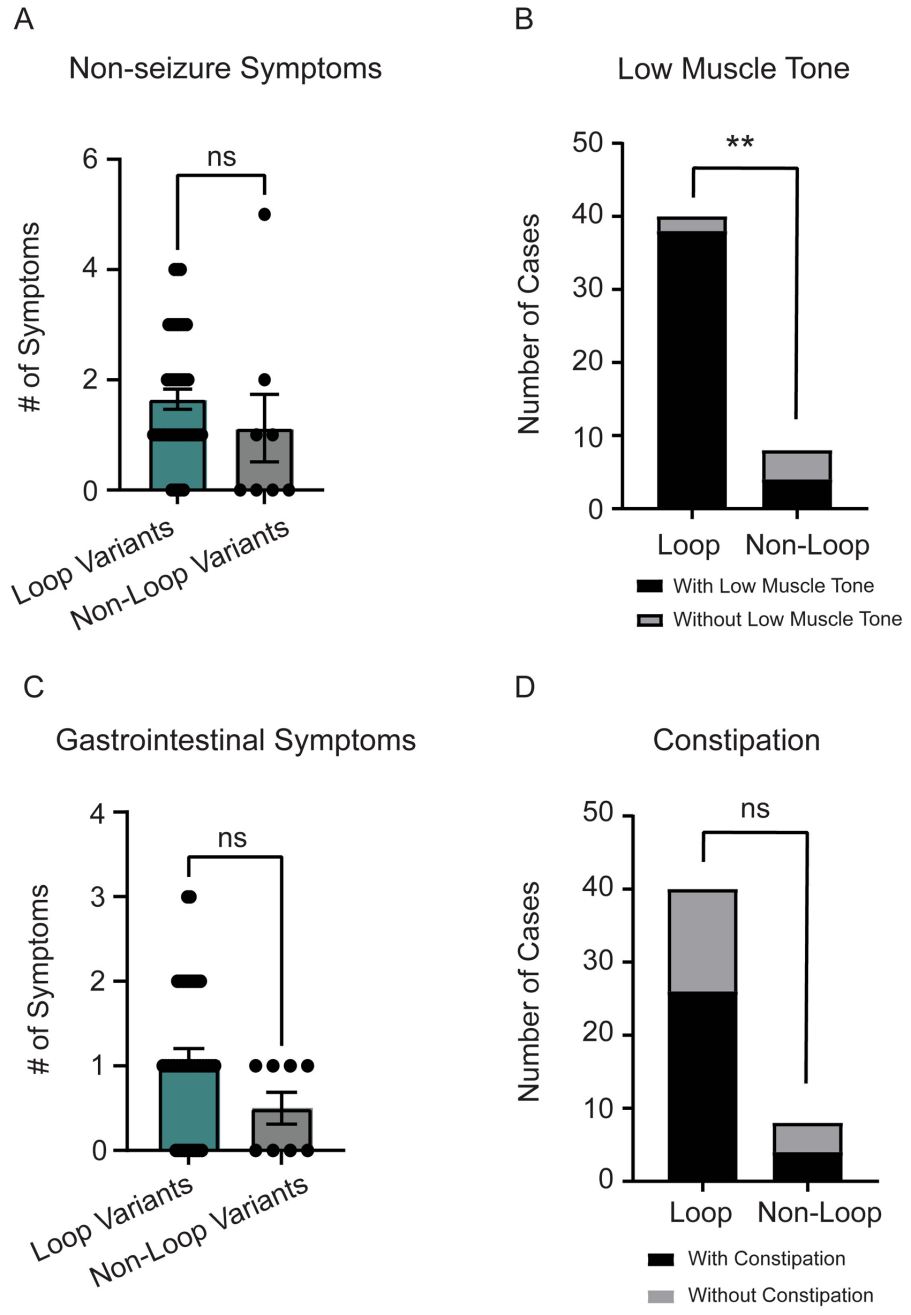
**(A)** Individuals with loop variants (*n* = 40) reported a higher number of non-seizure neurological symptoms compared to non-loop variants (*n* = 8) (Mann–Whitney test, *p* = 0.11). Reported non-seizure neurological symptoms included low-muscle tone, clumsy (poor motor coordination), movement disorder, cortical blindness, small head size (microcephaly), cerebral palsy, tic disorder, and high muscle tone (hypertonia). **(B)** The most reported non-seizure neurological symptom was low-muscle tone (*n* = 42). There was a significant trend for individuals with loop variants to have low muscle tone compared to non-loop variants (Fisher’s exact test, *p* = 0.0046). **(C)** Individuals with loop variants (*n* = 40) in *CSNK2A1* report a higher number of gastrointestinal (GI) symptoms compared to non-loop variants (*n* = 8) however, this difference was not statistically significant (Mann–Whitney Test, *p* = 0.09). GI symptoms included constipation, diarrhea, GERD, and celiac disease. **(D)** We did not observe a significant difference in the number of loop and non-loop individuals reporting constipation as an OCNDS symptom (Fisher’s exact test, *p* = 0.45). **p-value is less than 0.01.

Individuals with loop variants in *CSNK2A1* showed a trend toward a higher number of non-seizure neurological symptoms, though the difference was not statistically significant (*p* = 0.11, Figure 5A). The most common non-seizure neurological symptom was low muscle tone (*n* = 42) followed by small head size (microcephaly, *n* = 13). When comparing low muscle tone between loop (*n* = 44) vs. non-loop (*n* = 9) individuals, there was a significant difference wherein individuals with loop variants reported a higher frequency of low muscle tone symptoms (Figure 5B, *p* = 0.005, odds ratio 19 [95% CI: 2.896–110.6]). Of note, all individuals with variants predicted to disrupt ATP-binding (*n* = 11, Gly-rich loop) reported low muscle tone as a symptom and only 25% of individuals with variants in the N-terminal domain reported neurological symptoms (Supplementary Table S2). There was no significant difference between groups for reports of microcephaly (*n* = 13, 27% of cohort) (Supplementary Figure S2S, *p* = 0.42).

### Gastrointestinal symptoms

Individuals with loop variants in *CSNK2A1* reported more gastrointestinal (GI) symptoms, though the difference was not statistically significant (*p* = 0.09, Figure 5C). The most reported GI symptoms in our cohort were constipation (*n* = 30) and gastroesophageal reflux disease (GERD) (*n* = 9). Although there was not a significant difference between loop and non-loop variants in reports of constipation (*p* = 0.45), results suggest ~60% of patients report constipation symptoms regardless of variant location (Figure 5D). All 9 patients that reported GERD as a symptom had loop variants (Supplementary Figure S2O).

### Other OCNDS symptoms

Supplementary Figure S4 illustrates the percentage of participants that have each symptom with percentages as follows: % total patients in cohort with reported symptom (red), % of loop patients with symptom (teal), % of non-loop patients with symptom (black), and % of patients in the literature reported to have symptom (orange). No significant differences between loop and non-loop individuals were observed in the number of allergy (*p* = 0.57), autoimmune (*p* > 0.99), cardiac (*p* = 0.57), dermatological (*p* > 0.99), endocrine (*p* = 0.43), genitourinary (*p* = 0.40), infectious diseases (*p* = 0.95), orthopedic (*p* > 0.99), surgeries (*p* = 0.88), or joint (*p* > 0.99) symptoms reported (Supplementary Figure S2). While there was an observed trend for loop individuals reporting a higher visual symptom burden, this difference was not statistically significant (*p* = 0.11, Supplementary Figure S2J). The most reported visual symptom was astigmatism, which was also not statistically significantly different between groups (Supplementary Figure S2P). No significant differences were observed for the total number of reported seizure symptoms between loop and non-loop variants (*p* > 0.99, Supplementary Figure S2M). We were unable to analyze the comparison of age at seizure onset between groups due to missing data (Supplementary Figure S3F). Short stature (*n* = 22) and failure to thrive (*n* = 19) were the highest reported endocrine symptoms regardless of variant location. No significant difference between groups was observed for failure to thrive (Supplementary Figure S2Q, *p* = 0.45) nor for short stature (Supplementary Figure S2R, *p* = 0.26). Only individuals with loop variants reported genitourinary symptoms (Supplementary Figure S2F); notably, 5 individuals with loop variants (~24% of males in our cohort) reported undescended testicles (*data not shown*).

### Cognitive symptoms

**Figure 6:**
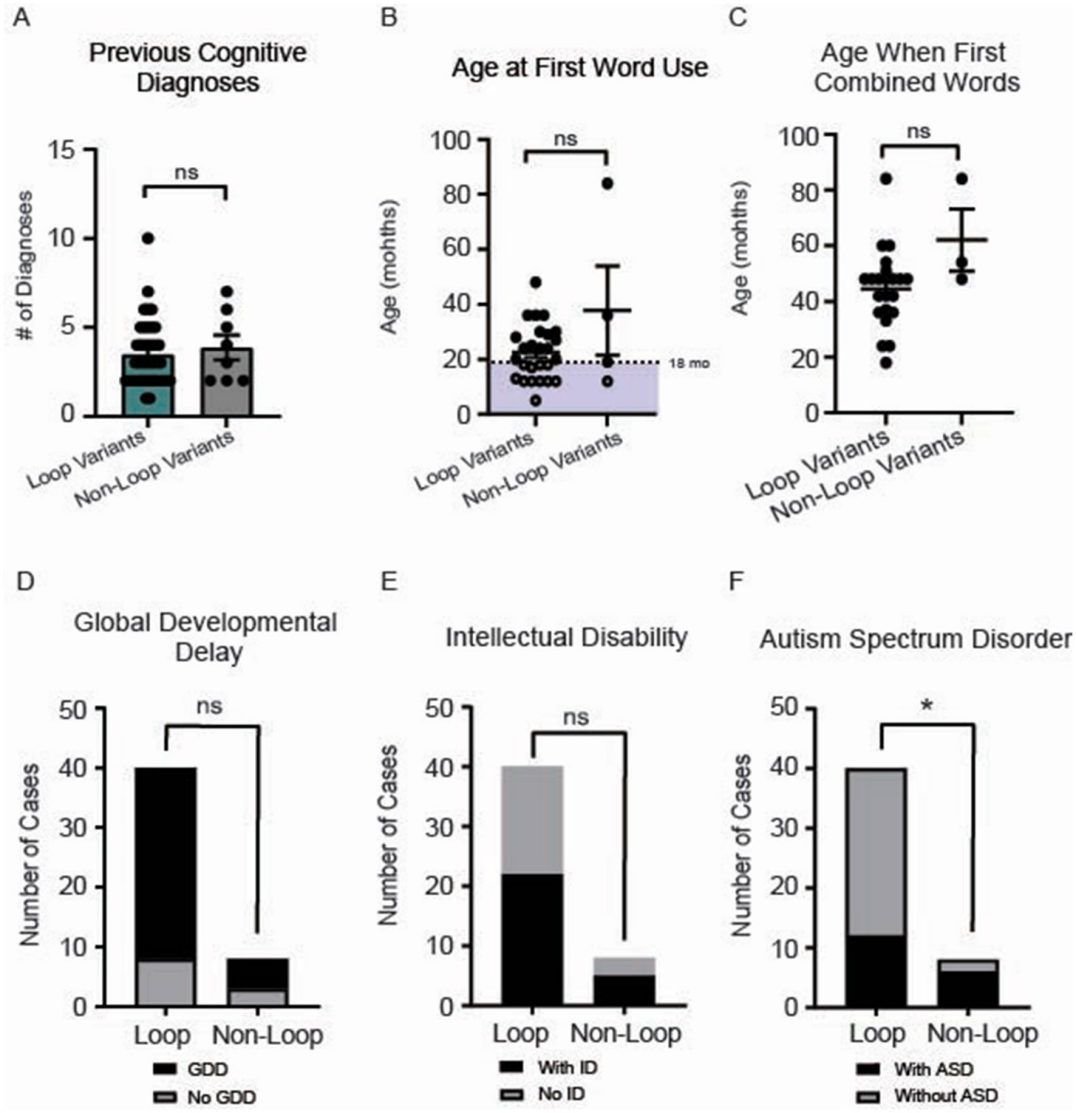
**(A)** No significant difference in the number of cognitive diagnoses was observed between loop and non-loop individuals (Mann–Whitney Test, *p* = 0.65). **(B)** Age at first word use was reported for 29 participants in our cohort, with no differences observed between groups (*p* = 0.80). Notably, an additional non-loop patient reported the use of first words after 7 years (*data not shown*). **(C)** Age at which individuals first combined words was reported for 23 individuals; no significant difference between loop and non-loop patients was observed (Mann–Whitney Test, *p* = 0.30). **(D)** No differences were observed in the frequency of reported global developmental delay (GDD) between groups (Fisher’s exact test, *p* = 0.36). **(E)** No differences were observed between groups for the prevalence of intellectual disability (ID) (Fisher’s exact test, *p* > 0.99). *p-value is less than 0.05. **(F)** We compared the frequency of autism-spectrum disorder (ASD) between loop and non-loop individuals; non-loop individuals were more likely to report ASD (6/8) than loop individuals (12/40) (Fisher’s exact test, *p* = 0.04).

No difference was observed for the number of cognitive diagnoses (*p* = 0.58) (Figure 6A). While initial word use typically emerges around 12 months of age in typically developing children, it can range up to 16 months. Because delayed speech beyond 18 months often prompts pediatric evaluation, we selected 18 months as our cutoff for assessing early language development (Feldman, 2019; I. Introduction, 1994). We present the age at first word use for 29 participants in our cohort, with no differences observed between groups (*p* = 0.8) (Figure 6B). Similarly, the ages at which individuals first combined words showed no significant difference between loop and non-loop patients (*p* = 0.30) (Figure 6C); notably, two additional individuals with loop mutations (at ages 22 and 45 months) and one non-loop individual (at age 43 months) reported “not yet” for combining words and one loop and one non-loop individual reported combining words after age 7 (*data not shown*). All patients in our cohort reported speech/language delays (Figure 2). We further assessed the number of patients that were non-verbal; all 4 patients had variants in loop regions. The frequency of global developmental delay and intellectual disability was not different between loop and non-loop individuals (Figure 6D, *p* = 0.36 and Figure 6E, *p* > 0.99). Since *CSNK2A1* is recognized as an autism-related gene with the highest gene score of 1 in the SFARI database,^2^ we compared the frequency of autism-spectrum disorder (ASD) between loop and non-loop individuals; non-loop individuals were more likely to report ASD (6/8) than loop individuals (13/40) (Figure 6F, *p* = 0.04). Additional milestones with no significant differences observed between groups were age at bowel control (*p* = 0.69), bladder control (*p* = 0.47), first crawled (*p* = 0.11), first walked (*p* = 0.41), and first sat up without support (*p* = 0.20) (Supplementary Figure S3).

**Figure 7:**
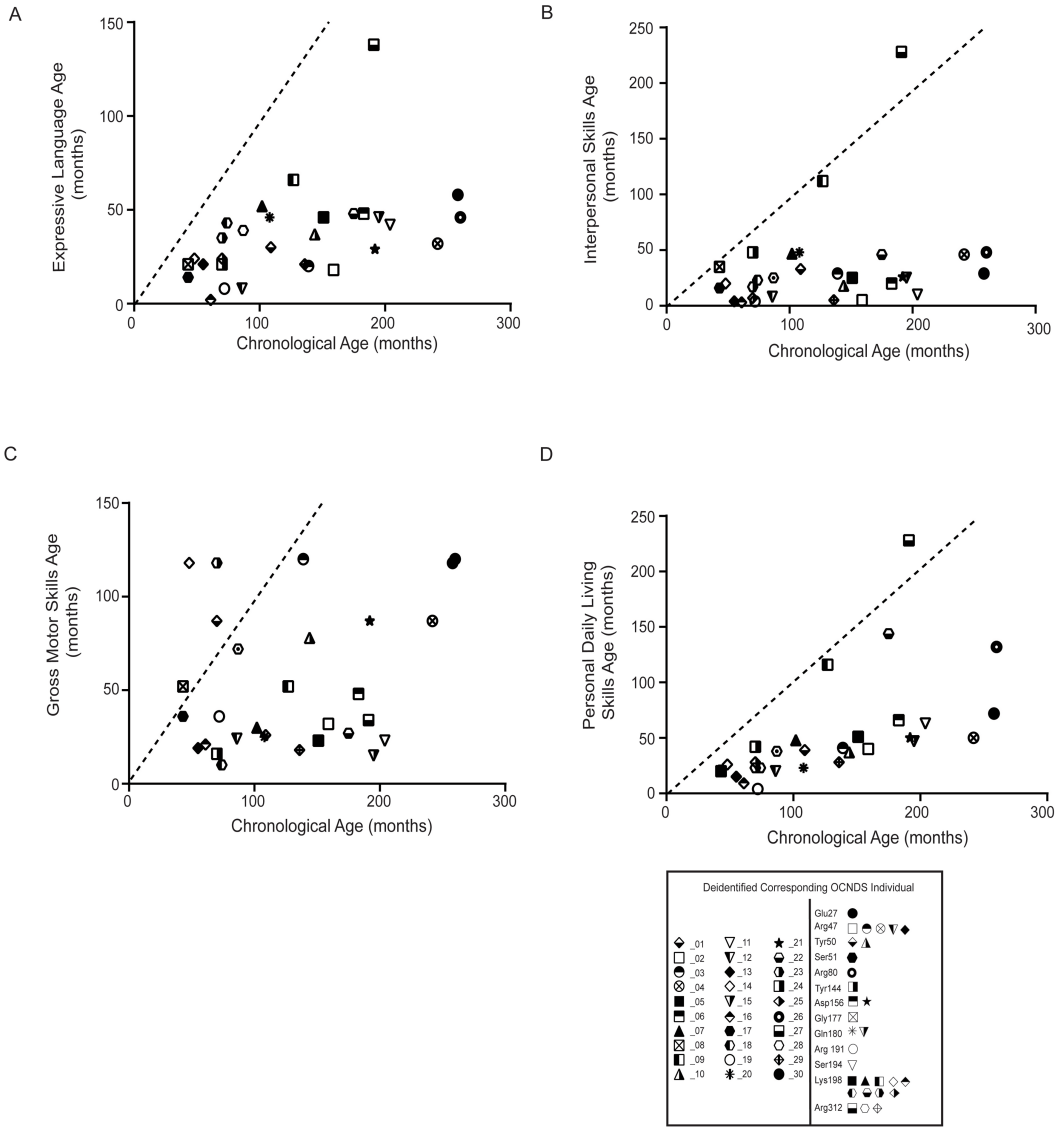
Statistical analysis of Vineland-III data was limited due to the small number of reported patients with variants in non-loop regions (*n* = 1–5) compared to those in loop regions. All plots compare the chronological age (x-axis) to the respective age at which adaptive behavior (y-axis label) is achieved; all plots reflect comparison to the population mean (dashed lines). Simple linear regression was used to assess whether OCNDS patients differ from the typical population in adaptive behavior skills. **(A)** Expressive communication subdomain age (in months) (y = 0.18, r^2^ = 0.22). **(B)** Interpersonal skills age (in months) (y = 0.17, r^2^ = 0.07). **(C)** Gross motor skills age (months) (y = 0.15, r^2^ = 0.06). **(D)** Daily living skills age (in months) (y = 0.41, r^2^ = 0.34). Legend at bottom depicts icons corresponding to the deidentified individual and their corresponding protein segment.

To further assess cognitive variables, we analyzed developmental patterns using responses to the Vineland Adaptive Behavior Scales, third edition (Vineland-III, Pearson Education Incorporated, San Antonio, Texas)^3^(Sparrow and Cicchetti, 1985). The average age of participants with Vineland-III responses was 128 months (~11 years). We observed that the majority of OCNDS patients in our cohort consistently scored below average for the typical developmental trajectory for the following subdomains: expressive language, interpersonal skills, gross motor skills, and personal daily living skills (Figure 7). Scatter plots were used to visualize Vineland-III scores for participants that had at least one completed entry for the following subdomains: expressive language (Figure 7A), interpersonal skills (Figure 7B), gross motor skills (Figure 7C), and personal daily living skills (Figure 7D). We compared the chronological age to the participants’ respective age at which they demonstrated adaptive behaviors. We observed that ~97% of OCNDS patients fall below the expected range for normal development for all categories except gross motor (~80% of patients below expected range) regardless of variant location. We observed an outlier across three domains (Figures 7A,B,D) that appears to demonstrate less developmental impairment compared to the rest of the cohort; this patient harbors a variant in the interdomain segment (Arg312Trp). We further compared a subset of domain and subdomain scores between loop and non-loop individuals (Supplementary Figure S5); for Adaptive Behavior Composite (ABC), Daily Living, Socialization, Communication, and Motor domains, most loop variants scored “low” or “moderately low.” Fewer Vineland reports were recorded for non-loop variants, however >50% reported “low” adaptive behavior across all domains. No significant differences were observed between groups for ABC (S5A, *p* = 0.83), daily living skills (S5B, *p* = 0.89), socialization skills (S5C, *p* = 0.73), communication skills (S5D, *p* = 0.82), and motor skills domains (S5E, *p* = 0.32). Similarly, fine (*p* = 0.20) and gross motor (*p* = 0.40) skills subdomains were not significantly different between groups (Supplementary Figure S5E).

### Sleep symptoms

**Figure 8:**
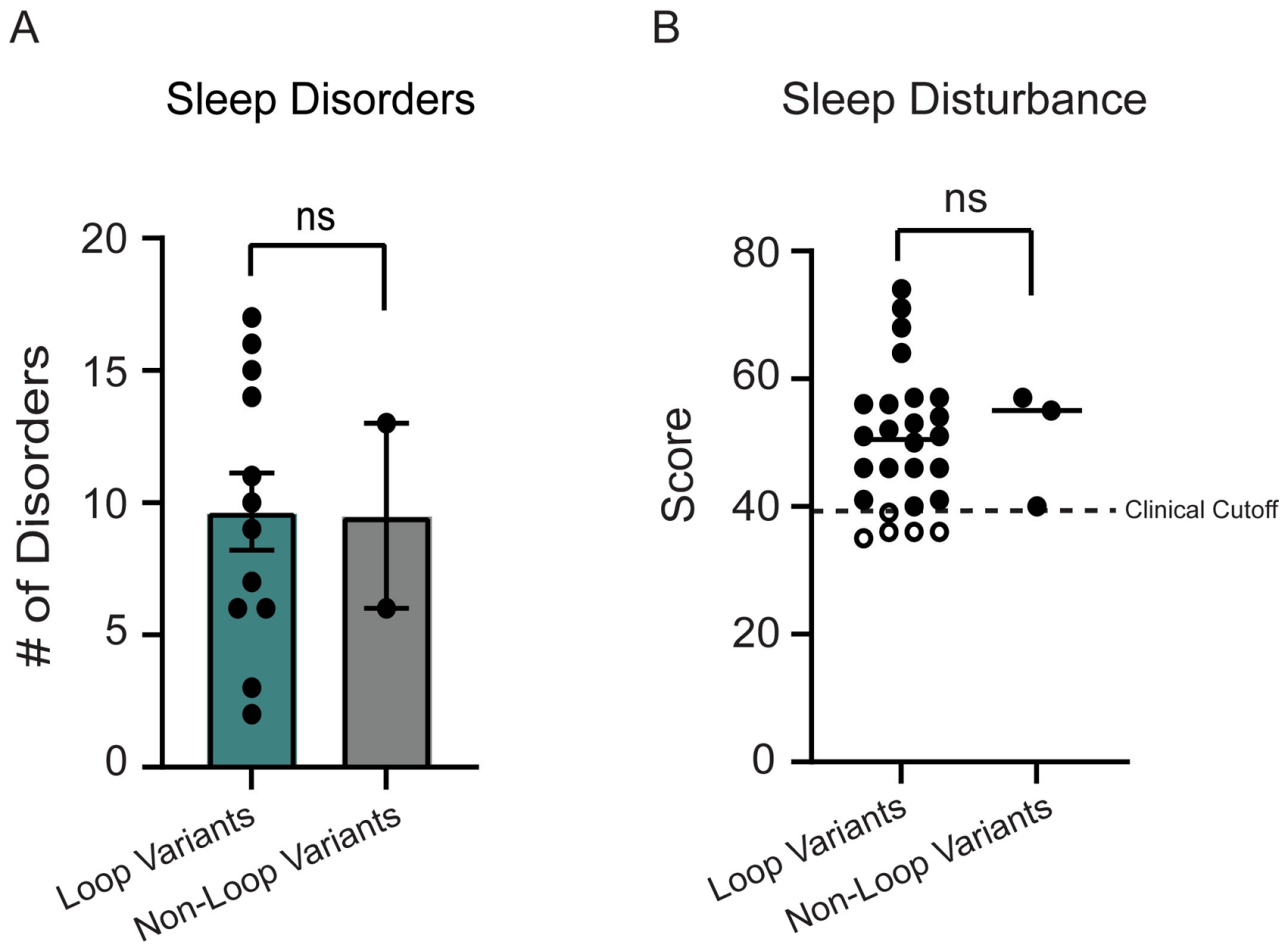
**(A)** 14 individuals reported sleep disorders in the SDB. No significant difference was observed between loop (*n* = 12) and non-loop (*n* = 2) individuals for the number of sleep disorders reported (Mann–Whitney test, *p* = 0.92). **(B)** The children’s sleep habits questionnaire (CSHQ) sleep disturbance scores were reported for 29 individuals. No significant differences were observed between loop (*n* = 26) and non-loop (*n* = 3) individuals (Mann–Whitney test, *p* = 0.72).

Since sleep disorders were reported as one of the top 3 most impactful symptoms in OCNDS via a 2021 Simons Searchlight Voice of the Community Report,^4^ we investigated the prevalence of sleep disorders and related symptoms in our dataset by analyzing the Sleep Disordered Breathing survey (SDB) and Children’s Sleep Habits Questionnaire (CSHQ). For the SDB, we plotted the total score, which is an index of the number of sleep disorders reported for a patient. The reference age of patients who reported sleep symptoms ranged from 2 to 12 years of age, with a median age of 8.5 years. No significant differences between individuals with non-loop (*n* = 3) and loop (*n* = 26) variants were observed for total number of reported sleep disorders (Figure 8A, *p* = 0.92). The CSHQ has a clinical cutoff for pediatric sleep disorders at the index score of 39, meaning that scores 39 or above indicate a pediatric sleep disorder (Owens et al., 2000). We did not observe a significant difference between the index score in loop vs. non-loop (*p* = 0.72, Figure 8B). It is notable that ~83% of patients in our cohort meet the clinical cutoff for pediatric sleep disorders.

### Medication use

For a subset of participants (*n* = 34, 29 loop, 5 non-loop), we had data reported on medication use. We observed a non-significant trend where individuals with loop variants reported higher medication numbers (*p* = 0.29) (Supplementary Figure S2N). Both loop and non-loop individuals reported medication use across various classes, with the most reported medications being melatonin for sleep (*n* = 10, 34%; 8 loop, 2 non-loop) and polyethylene glycol for constipation (*n* = 8, 28%, 7 loop, 1 non-loop).

### Comparing CK2β-binding variants

**Figure 9:**
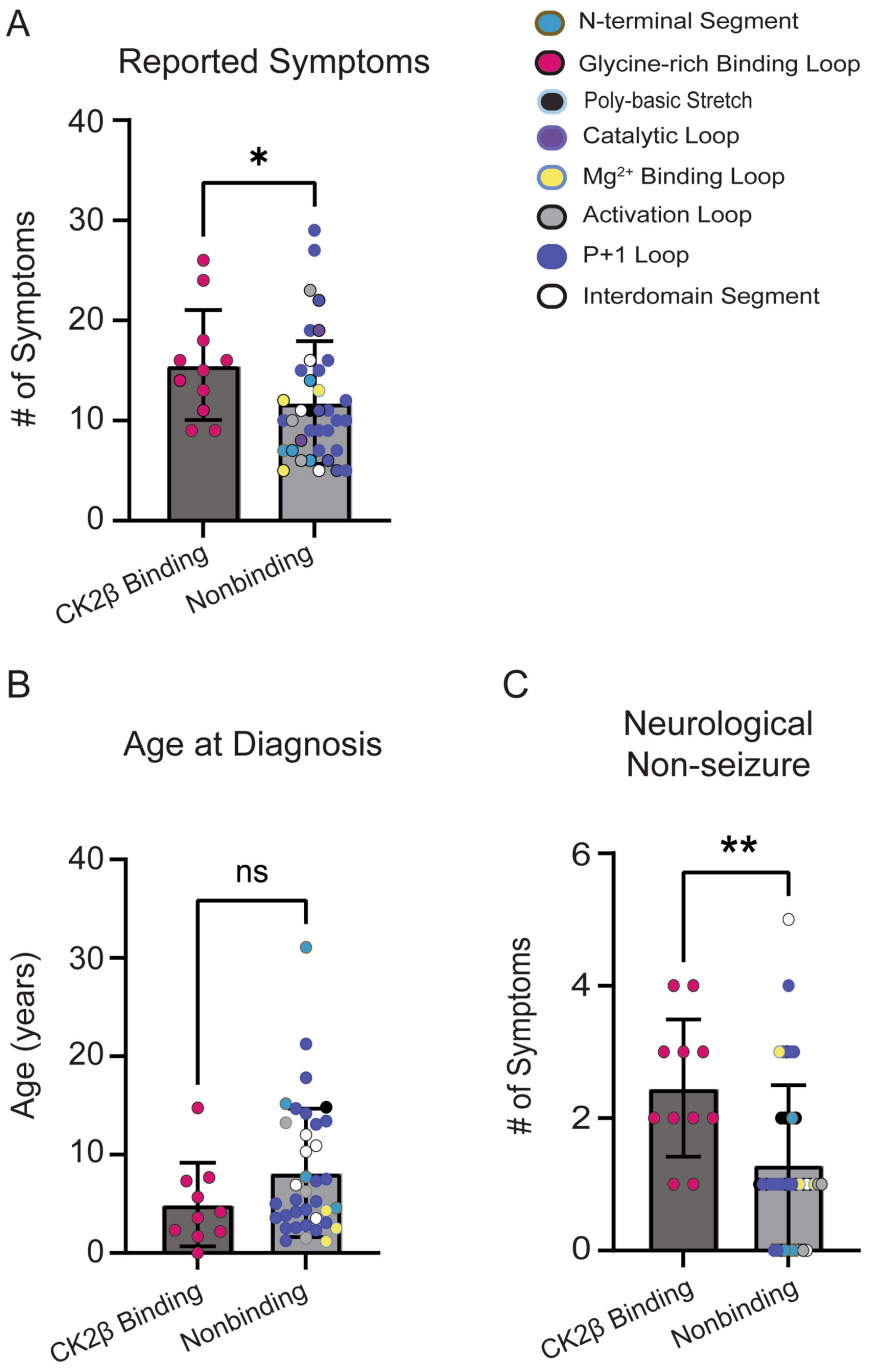
**(A)** CK2β-binding variants reported a higher number of symptoms than nonbinding variants (Mann–Whitney test, *p* = 0.03). Colored dots on plot correlate to the assigned colors representing CK2α protein segments. **(B)** No significant difference was observed for age at OCNDS diagnosis between CK2β-binding and nonbinding variants (Mann–Whitney test, *p* = 0.14). **(C)** Non-seizure neurological symptoms were more prevalent in CK2β-binding variants (*p* = 0.003). *= *p*-value is less than 0.05; ** = *p*-value is less than 0.01.

The residues in CK2α’s Gly-rich loop are important for ATP binding and catalysis and bind to CK2β (Unni et al., 2022). Given the role of CK2β in regulating CK2’s activity and the high symptom count for individuals with variants in the Gly-rich loop, we repeated a subset of analyses comparing individuals with variants at amino acid residues that bind CK2β (*n* = 11; residues 36–73, 103–108) to individuals with all other missense variants (*n* = 37). CK2β-binding variants exhibited a higher symptom burden (Figure 9A, *p* = 0.04). While we observed a trend for CK2B-binding variants reporting an earlier age at diagnosis (Figure 9B), this difference was not statistically significant (*p* = 0.14). Individuals with CK2B-binding variants reported a higher number of neurological (non-seizure) symptoms (Figure 9C). Our analyses yielded similar results as our loop vs. non-loop comparisons, with no differences between the total number of reported symptoms between groups in the following categories: endocrine (*p* = 0.92), dermatological (*p* > 0.99), genitourinary (*p* = 0.40), gastrointestinal (*p* = 0.80), seizures (*p* = 0.93), surgeries (*p* = 0.63), and visual (*p* = 0.07) (Supplementary Figure S6), with visual symptoms showing a non-significant trend for a higher symptom burden in CK2β-binding variants.

To further evaluate phenotypic variability among individuals with *CSNK2A1* variants published in the literature, we compiled all published cases into a separate table (Supplementary Table S3). This table includes individuals reported in the literature and presents a set of symptom categories based on the level of clinical detail provided. Each row represents a reported case, annotated by the presence or absence of symptoms across key domains. While this enables side-by-side comparisons of individuals with the same variant, differences in reporting detail and terminology across sources limited our ability to identify trends relative to our cohort data. Consistent with our cohort, we observed a high frequency of neurological symptoms, speech delay/disorders, and global developmental delay. Together, these results suggest the need for additional genotype–phenotype analyses to improve our understanding of symptom presentation across the lifespan of individuals with OCNDS. Further studies are needed to ascertain functional changes that particular mutations exert on the protein which may help explain why individuals with specific variants present with a higher symptom burden. Increased participation by OCNDS families in Simons Searchlight will facilitate more in-depth analyses of mutations across CK2α segments and eventually comparisons by amino acid residue or specific variants. This could yield supportive information to assist families in predicting overall symptom burden and severity over the patient’s lifespan. In the future, this may also support tailored therapeutic approaches dependent on mutation location.

## Discussion

OCNDS presents with a wide array of clinical manifestations at varying severity levels including intellectual disability, speech and motor delays, behavioral abnormalities, disrupted sleep patterns, seizures, and growth abnormalities (Chung and Okur, 2022), similar to many other neurodevelopmental disorders. Therapeutic management of OCNDS includes many therapies including feeding, physical, occupational, and standard supportive developmental therapies (Chung and Okur, 2022). Studies of other neurodevelopmental disorders emphasize the importance of early detection to enable early interventions. One example is in tuberous sclerosis complex where early intervention has been shown to improve cognitive outcomes (Northrup et al., 2021; Słowińska et al., 2018) and where a lack of early intervention has led to severe complications (Gipson, 2018; Lim et al., 2022). As the estimated prevalence of OCNDS is 1 in 100,000 individuals (Gillentine et al., 2022), we recognize that OCNDS is underdiagnosed and expect to see the number of identified patients increase with improved access to genetic testing. In our cohort, we limited our analysis to missense variants thus, not all CK2α protein segments were represented. However, increased genetic testing can enhance our understanding of whether variant location within a gene can predict symptom number, type, and/or severity.

### OCNDS phenotypic presentation

Prior studies have focused on individual case reports or small patient cohorts (Akahira-Azuma et al., 2018; Chiu et al., 2018; Chung and Okur, 2022; Jafari Khamirani et al., 2022; Martinez-Monseny et al., 2020; Nakashima et al., 2019; Nan et al., 2024; Okur et al., 2016; Ramadesikan et al., 2024; Zhuri et al., 2024) while our study provides a larger sample size (*n* = 48 individuals). We compared missense variants in conserved loop regions of CK2α to other reported missense variants in Simons Searchlight. We observed that individuals with missense variants in CK2α protein segments with loop structures presented with an increased prevalence of low muscle tone, were diagnosed at earlier ages, and exhibited a trend of a higher symptom count than individuals with variants in non-loop segments (Figures 4B,C, 5B). We acknowledge that an individual with a higher symptomatic burden may visit a clinician more frequently than an individual with fewer symptoms; however, this highlights the need for careful considerations of symptom presentation at an early age and the significance of access to early genetic testing when symptoms first arise such as delayed milestones. For example, a recent paper analyzing Vineland-II motor standard scores in neurodevelopmental syndromes included OCNDS patients (Santana Almansa et al., 2024) and observed that individuals with OCNDS exhibited an average age of walking independently at 30.6 months compared to 18 months [age at which ≥75% of children would be expected to achieve walking independently according to the American Academy of Pediatrics (Zubler et al., 2022)]. Our data support this delayed milestone with an average walking age of 24 and 26 months for individuals with loop and non-loop variants, respectively (Supplementary Figure S3D). This highlights an observable delay in motor abilities in OCNDS patients that may be noticed at regular pediatric visits and potentially trigger genetic testing.

Consistent with previous reports (Chiu et al., 2018; Nakashima et al., 2019; Ramadesikan et al., 2024; Wu et al., 2021), neurological symptoms were one of the most reported OCNDS symptom categories in our cohort however, our study highlights speech and language delay/disorders as a key feature of OCNDS, with all patients reporting this symptom (Figure 2). The measure of age at first word use further illustrates the severity of this symptom; 60% of participants with reported age did not produce their first words until after 18 months (Figure 6B) and some individuals had not yet produced words. Individuals with variants in loop regions were more likely to report low muscle tone (hypotonia) as a symptom (Figure 5B). We observed short stature as the highest reported endocrine related symptom regardless of variant location, present in ~46% of our cohort (Supplementary Figure S2R). We acknowledge that reporting of symptoms such as short stature represents a snapshot at a specific age. Future studies are necessary to analyze growth charts over time to improve our understanding of this phenotype.

### Vineland

The Vineland-III is used to explore metrics on cognitive, behavioral, and daily functioning and the scores provide insights into how an individual functions in everyday life compared to others of the same age (Dang Do et al., 2022; Patel et al., 2024). We analyzed adaptive behavior composite (ABC), daily living skills, socialization, communication and motor domains (Supplementary Figure S5). We did not observe any clear trends between loop and non-loop variants; however, we found that the majority of OCNDS patients scored “low” in adaptive behavior, regardless of variant location. Additionally, ~97% of OCNDS patients fell below the expected range for normal development for expressive language, interpersonal skills, and personal daily living skills regardless of variant location (Figure 7). Additional datasets could establish a more accurate progression timeline and assist in creating tailored intervention plans for managing OCNDS symptoms.

### Sleep

Previous reports show that ~86% of patients with neurodevelopmental disorders present with sleep disorders (Shelton and Malow, 2021; Williams Buckley et al., 2020). Case reports of OCNDS patients describe issues with sleep apnea, nocturnal awakening, and prominent displays of fatigue (e.g., falling asleep during evaluations) (Chiu et al., 2018; Ramadesikan et al., 2024; Trinh et al., 2017; Wu et al., 2021). The SDB and CSHQ surveys were evaluated to determine the prevalence and signatures of sleep disturbance in patients with OCNDS across genotypes. In our dataset, 83% of patients reported sleep disturbances that met the clinical cutoff for pediatric sleep disorders (Figure 8B). Previous research on sleep disorders observed in other clinical populations suggest that sleep disorders may be a secondary effect of other health-related consequences of disorders (e.g., seizure prevalence, hypotonia, gastrointestinal issues) (Al Lihabi, 2023; Halstead et al., 2021; Shelton and Malow, 2021). Therefore, managing these symptoms could influence sleep efficiency and potentially improve behavioral outcomes. A potential future assessment could be fitness tracker bands (e.g., rings or other wearable equipment) worn by patients (Lee et al., 2023; Miller et al., 2020; Robbins et al., 2024). Using such tools could lead to a more comprehensive understanding of how sleep patterns and sleep duration are affected in OCNDS (DiJoseph et al., 2021; Furia et al., 2024; Miller et al., 2020).

### CK2α structural changes as potential contributors to phenotypic presentation

The p + 1 loop and Gly-rich loops exhibited the highest mutational burden with 2.1 and 1.83 patients per amino acid residue, respectively (Table 1). This was not surprising since pathogenic variants in neurodevelopmental disorders often occur in highly conserved protein regions (Geisheker et al., 2017). There is evidence of CK2α structural changes in the presence of specific mutations that may contribute to why certain mutations present with a more severe OCNDS phenotype. For example, the Glycine residues in the Gly-rich loop (amino acid sequence Gly46-Arg47-Gly48-Lys49-Tyr50-Ser51-Glu52-Val53) permit close proximity of the loop backbone and the *β*- and *γ*- phosphates of ATP/GTP (Bossemeyer, 1994; Niefind et al., 2001, 2009; Niefind and Battistutta, 2013). Structural studies demonstrated that replacing glycine-48 with aspartic acid (G48D) led to improved kinetic properties for ATP, possibly due to a quicker release of ADP from the ATP pocket due to steric and electrostatic effects (Chaillot et al., 2000). However, when substituted with a positively charged amino acid (G48K), the protein exhibited an electrostatic barrier that decreased the kinetic properties for ATP. This may explain some of the phenotypic differences in patients with different amino acid substitutions at the same residue. Previous *in vitro* work using skin fibroblasts collected from patients with Arg47Gly/Gln variants tested kinase activity of the mutants compared to wild type (WT): Arg47Gly had 20–30% reduced kinase activity, and Arg47Gln demonstrated 40–50% reduced kinase activity, however only one consensus peptide was utilized in this study limiting interpretation (Dominguez et al., 2021a). Structural studies have suggested that the Gly-rich loop may collapse in an inactive structure such that Arg47 blocks the active ATP-binding site (Raaf et al., 2009). Furthermore, the entire Gly-rich binding loop binds to CK2β; thus, it is possible that some variants affect the binding of CK2α to its CK2β counterpart (Unni et al., 2022). Thus, disruptions to CK2β’s regulatory function may help explain why variants in this region have displayed the widest range of phenotypes in OCNDS patients (Wu et al., 2021).

Specific residues in the p + 1 loop (Arg191, Arg195, and Lys198) are important for substrate recognition (Sarno et al., 1999; Unni et al., 2022). Alterations in this region are proposed to alter the formation of regulatory holoenzyme oligomers (Unni et al., 2022) and are implicated in the mechanism where the N-terminal domain of the β-subunit downregulates CK2 activity (Sarno et al., 1997). Functional analyses of the K198R mutant illustrated a 20–30% reduction in activity as compared to WT (Dominguez et al., 2021a) while structural analyses indicated a significant shift in the sulfate ion that marks the anion binding site in this position (Werner et al., 2022). This may explain previous observations of altered substrate specificity when K198R was expressed in *E. coli* and exhibited a decreased preference for acidic resides in the P + 1 position, decreased preference for threonine phosphorylation, and an increased preference for tyrosine phosphorylation (Werner et al., 2022). Additional structural predictions have been made for other *CSNK2A1* variants (see Unni et al., 2022). We grouped individuals based on known or predicted protein-level effects from Unni et al. (2022) and observed that loop variants were more often associated with disrupted substrate or ATP binding, while non-loop variants tended to affect folding or localization (Figure 4D and Supplementary Figure S2). Although individuals with substrate or ATP-binding disruptions showed a trend toward greater symptom burden (Figure 4D), future studies with increased patients across functional categories are necessary to determine whether these differences have clinical relevance.

### Mechanisms and considerations for OCNDS therapeutic development

In a 2021 OCNDS “Voice of the Community” survey administered by Simons Searchlight, the top 3 most impactful symptoms were language delay/inability to speak, intellectual disability/developmental delay, and sleep issues.^5^ Our data highlight that 100% of our cohort report speech/language delay, ~77% report global developmental delay, and ~82% meet the clinical cutoff for sleep disorders. Additionally, our dataset showed that ~63% of individuals reported constipation. These symptoms stand out as common therapeutic focus areas in OCNDS regardless of variant location. Therefore, our study suggests a core set of OCNDS symptoms.

CK2β is responsible for maintaining the stability of the CK2 tetramer (α2β2) and it modulates substrate specificity, meaning it helps determine which proteins CK2 will phosphorylate (Unni et al., 2022). Given the important role of CK2β, we analyzed our cohort to compare variants in residues that normally bind CK2β vs. all other variants. We observed that variants in CK2β-binding residues reported a higher number of symptoms and a lower age at OCNDS diagnosis (Figures 9A,B). Of note, another neurodevelopmental syndrome called Poirier-Bienvenu neurodevelopmental syndrome (POBINDS) exists resulting from mutations in the *CSNK2B* gene, which encodes for the CK2β protein. POBINDS and OCNDS share many symptoms including developmental delay, behavioral problems, intellectual disability, hypotonia, and language/speech delays however, seizures are a more common symptom in POBINDS (~90%) as compared to OCNDS (~30%) (Ballardin et al., 2022; Lippa et al., 2024). It is currently unclear whether OCNDS and POBINDS have common mechanistic components that could be shared targets for therapeutic development.

### Limitations and future directions

We were limited by the number of individuals enrolled in Simons Searchlight. As such, our findings may not fully capture the phenotypic spectrum associated with all *CSNK2A1* variants. A key limitation of this study is that our analysis was restricted to individuals with missense variants in *CSNK2A1*. While this reflects the genetic distribution in our dataset—where ~95% of individuals with available medical history data carried missense variants; other variant types without confounding variables such as deletions, splice site, nonsense, and frameshift variants were underrepresented (*n* = 1–2 per category, Supplementary Table S1) and the majority lacked sufficient medical history data to support inclusion. Furthermore, these non-missense variants are presumed to result in loss-of-function and may follow different molecular mechanisms compared to the region-specific functional effects hypothesized for missense variants. Deletions often span multiple protein domains or neighboring genes, making them less suitable for region-based analyses. Additional investigation into other variant types in *CSNK2A1* is warranted. A recent literature review noted that individuals harboring *CSNK2A1* null variants presented with a milder phenotype than individuals with missense variants, specifically annotating a reduced frequency of symptoms associated with dysmorphic facial features, language deficits, and intellectual disability (Nan et al., 2024). We did not have sufficient representation of null variants in the dataset to investigate this however, the subset we assessed more closely aligned with non-loop variants (Supplementary Figure S1) and had fewer total symptoms, similar to these previous reports. This difference leads to questions of pathogenic mechanisms in OCNDS—if individuals with null variants (i.e., a haploinsufficiency model) have a milder phenotypic presentation, it is possible that missense variants may operate via a different mechanism (e.g., gain-of-function, dominant negative, or neomorphic mechanism). The presence of both gain-of-function (GOF) and loss-of-function (LOF) mechanisms within the same gene have been observed across many rare neurodevelopmental disorders, including developmental and epileptic encephalopathies (DEEs) (Blanchard et al., 2015; Jiang et al., 2019; Sanders et al., 2018). Interrogating these differences is crucial to therapeutic advancements as distinct mechanisms may require distinct approaches especially when considering gene modification therapies (e.g., ASOs, gene therapy). Experiments to model CK2 protein structure and localization in the presence of specific mutations would yield important insights into disease mechanisms and subsequently help determine feasible therapeutic approaches. It has already been shown that protein subcellular localization is altered in the presence of certain variants (Dominguez et al., 2021b), which could affect overall function. Future experiments exploring structure, location, and substrate specificity of CK2 under different mutational contexts should include representative variants from all protein segments to enhance our knowledge of potential regional effects.

While our analysis initially grouped variants by their location within conserved loop structures, we recognize that this categorization may reflect an underlying enrichment of pathogenic variants in functionally critical domains, rather than a specific effect of loop-based secondary structure. Many of the variants classified within “loop” regions are located in highly conserved sites essential for kinase function—such as ATP binding, substrate recognition, or holoenzyme assembly—whereas most VUS and loss-of-function (LOF) variants fall outside these regions and lack functional validation. We acknowledge that the small sample size limits the statistical power of these comparisons and may overestimate effect sizes. Future studies with larger cohorts and more comprehensive functional annotations will be essential to assess whether specific phenotypic patterns correspond more closely with disruptions in catalytic activity, substrate affinity, or protein folding, rather than structural classification alone.

Simons Searchlight data is reported by caregivers of individuals with OCNDS. Therefore, we are limited by the questions asked of caregivers within the surveys, the memory recall of caregivers spanning many years of symptoms and treatments, and the data release dates from SFARI. An option for future studies would be to analyze medical records of OCNDS patients. The CSNK2A1 Foundation is currently partnering with Citizen Health to pursue this type of analysis (Rushing and Sills, 2024). Furthermore, we cannot rule out the possibility that the patients in this cohort may have already been reported in the literature. To facilitate potential comparisons, we generated Supplementary Table S3 which summarizes the presence or absence of symptoms across organ systems in existing published cases. However, direct comparisons to our cohort were limited by variability in the level of detail and completeness of symptom reporting across publications.

In this study, we used total symptom count as a proxy for “symptom burden” across individuals with OCNDS. While this cumulative count offers a useful metric for comparing the breadth of clinical involvement, it does not directly reflect the severity or functional impact of individual symptoms. For example, a participant with a single severe symptom (e.g., intractable epilepsy) may experience greater daily impairment than one with multiple mild symptoms. As emphasized in a recent genotype–phenotype comparison paper (Nagy et al., 2022), integrating phenotypic severity scores into genotype–phenotype analyses can enable more detailed interpretation of clinical impact. At present, our dataset lacks standardized severity ratings within each symptom domain, which limits our ability to assess how symptom burden correlates with overall disease severity. In many cases, only the presence or absence of a symptom could be recorded, without clinician-rated severity assessments or functional measures. As a result, symptom count in our study should be interpreted as an indicator of multisystem involvement rather than a comprehensive measure of clinical severity. We acknowledge this limitation and underscore the importance of incorporating validated severity metrics in future studies to better capture the full spectrum of disease impact and clarify genotype–phenotype relationships in OCNDS.

## Methods

### Study design and ethics approval

We conducted a retrospective review of OCNDS natural history data collected via Simons Searchlight. Salus IRB reviewed the study entitled “Analyzing OCNDS Data from Simons Searchlight Natural History Study” and determined that the activities do not constitute regulated research involving human subjects (Study ID 23134–01). STROBE guidelines were followed when preparing the article.

### Data processing

This study did not involve direct clinical examinations. Natural history data was collected through Simons Searchlight. Participants in Simons Searchlight are recruited from the CSNK2A1 Foundation and SFARI. The initial size of Simons Searchlight *CSNK2A1* Dataset v12.0 (*n* = 220) was filtered to only include reports with confirmed genetic status (*n* = 90) followed by filtering for reports with a listed c- or p-nomenclature, then missense variants, then classification as likely pathogenic or pathogenic, and lastly, removing participants that had additional genetic variants that could contribute to their symptom presentation. Of the participants that met the qualifications, SFARI_IDs (anonymous patient identifiers) were correlated across the following surveys (names align to data export names from Simons Searchlight): “Children’s Sleep Habits Questionnaire (CSHQ),” “Lab Results,” “Sleep and Disordered Breathing,” “Seizure History,” “Seizure Survey 2,” “Previous Diagnoses,” “Simons Variation in Individuals Project (SVIP) Medical History Interview,” “SVIP medications Interview,” “Background History Questionnaire (BGHX),” and “Vineland-III.”

Subsequent analyses further classified participants according to variant location in either loop regions (i.e., Glycine-rich binding loop, catalytic loop, Mg^2+^-binding Loop, activation loop, p + 1 Loop) or non-loop regions (i.e., N-terminal segment, poly-basic stretch, Hinge + Helix αD, interdomain segment, C-terminal segment). We cross-referenced the amino acid sequence with existing literature to identify the specific CK2α protein segments and assign individual mutations to the designated segment. Using the Simons Searchlight “svip_mhi_summary” (Simons Variants in Individuals Project [SVIP]) medical history interview, we generated pivot tables in Microsoft Excel specific to each phenotypic comparison (e.g., organ system, ages, survey scores). The same methodology was used when comparing variants in residues that bind CK2β to non-binding residues.

### Statistical analysis

GraphPad PRISM 10 software was used for all statistical analyses and reviewed via consultation with the Vanderbilt Biostatistics Clinic. No power analyses were conducted. For comparisons of loop vs. non-loop variants, Mann–Whitney tests were utilized for continuous variables and Fisher’s exact tests for categorical variables (presence vs. absence). Data were presented as means ± SEM. The authors thank the Biostatistics Clinics operated by the Department of Biostatistics, Vanderbilt University School of Medicine and sponsored by the Vanderbilt institute for Clinical and Translational Research, funded by CTSA award no. UL1 TR002243 from the National Center for Advancing Translational Sciences. Symbols used in figures are defined as such: *p < 0.05; **p < 0.01; ***p < 0.001; n.s. = not significant.

### Settings, participants, and data sources

OCNDS natural history data was obtained from the Simons Foundation Autism Research Initiative (SFARI) program Simons Searchlight.^6^ Simons Searchlight is an online international research program, building a natural history database and resource network for over 175 rare genetic neurodevelopmental disorders. Data was collected via standardized online surveys. Data export was Version 12.0 (v12.0), which included data from May 2016 to October 2023.

#### Vineland-III

The Vineland Adaptive Behavior Scales, Third Edition (VABS-3) is a standardized tool to assess adaptive functioning level overall and across domains of communication, daily living skills, socialization, motor skills, and maladaptive behaviors, each consisting of further subdomains. It is validated from birth to 90 years of age. Age of individuals in the Vineland-III dataset ranged from 2.5–21.5 years of age. According to the publisher’s guide, the normative mean for domain scores is 100 with a standard deviation of 15, with higher scores indicating better adaptive behavior. Domains assessed are Adaptive Behavior Composite (ABC), Communication, Socialization, Motor, and Daily Living Skills (DLS) (Pollak et al., 2023). For each domain, a standard score is given with a higher number reflecting stronger adaptive behavior. The ABC score is based on the three specific adaptive behavior domains: communication, daily living, and socialization; it provides an overall summary of the individual’s adaptive functioning. The daily living skills domain measures an individual’s performance on everyday tasks appropriate for that patient’s age; this domain standard score is derived from subdomain scores on personal, domestic, and community measures. The socialization domain assesses an individual’s functioning in social situations, based on the following subdomains: interpersonal relationships, play and leisure, and coping skills. The communication domain reflects an individual’s capacity to exchange information with others, which is based off the following subdomains: receptive communication, expressive communication, and written communication. The motor domain measures an individual’s use of gross and fine motor skills in their daily life. Gross motor skills assess the use of arms and legs for movement and coordination while fine motor measures skills tests manipulation of objects using hands and fingers. For patients that had more than one entry, the most recent report was used for analysis.

#### Sleep Assessments

Sleep phenotypes were evaluated with two caregiver‑reported instruments administered through Simons Searchlight: the Children’s Sleep Habits Questionnaire (CSHQ) and Sleep & Disordered Breathing (SDB) Checklist. The CSHQ assesses sleep-related behaviors in patients and rates each item on a scale that addresses key clinical sleep complaints commonly associated with sleep disorders. The scale consists of 33 items to investigate children’s sleep habits and sleep-related problems. It has eight subscales that screen children’s sleep disorders including resistance to bedtime, difficulty in falling asleep, sleep duration, sleep anxiety, night waking, parasomnia, sleep problems related to respiration, and daytime sleepiness. The total test score is calculated by summing the scores obtained from the items. Values above 39 are considered clinically significant (Owens et al., 2000). The SDB checklist is a 22‑item yes/no checklist that screens for specific night‑time breathing or arousal events (e.g., snoring, apnea, gasping). Affirmative responses (coded 1) were summed to create an individual sleep‑symptom count (range 0–22), representing the number of distinct sleep‑related disturbances reported for each participant.

#### Bias

We observed that the number of individuals with mutations in CK2α non-loop segments was lower than individuals with mutations in loop segments. Due to the retrospective study design, we were limited by existing participants in Simons Searchlight at the time of data export. Thus, there may be selection bias (e.g., participating families may be more symptomatic than others or families may have chosen not to participate based on limited available languages for the medical history intake). Additionally, as the data was reported by caregivers, there is a possibility of recall bias.

## Conclusion

There is limited understanding of genotype–phenotype correlations in OCNDS. This study represents the largest OCNDS cohort analyzed for phenotypic variability to date. Kinases have many highly conserved domains and as CK2 is a critical kinase in many biological processes, we aimed to explore how mutations across conserved genetic regions in *CSNK2A1* may impact phenotype presentation in OCNDS. In this publication, we show that individuals with mutations in conserved loop regions of CK2α present with a higher frequency of low muscle tone symptoms and are diagnosed at a younger age than individuals with mutations in other protein segments. Additionally, we identified a statistically significant increase in overall symptom burden and neurological (non-seizure) symptoms among individuals with mutations in the glycine-rich loop, which is critical for binding both the CK2 beta subunit (CK2β) and ATP. Our results expand on previous genotype–phenotype analyses and highlight the need for additional studies to ascertain functional changes that particular mutations exert on the protein. Given that no phenotypic differences were observed between variants for speech and language delay/disorders, constipation, sleep, global developmental delay, or intellectual disability, this suggests a set of core OCNDS symptoms that can guide therapeutic development and benefit patients regardless of mutation location. With recurrent longitudinal data expected to become available over time, we hope that collaborative efforts will enhance rare disease research on *CSNK2A1* variants and the development of preclinical models, paving the way for future therapeutic trials. Furthermore, with an increased sample size in future studies, we can better assess whether specific symptom categories are more frequent in specific mutational contexts. We acknowledge there may be other unidentified or underrepresented *CSNK2A1* variants; with increased access and utility of sequencing, additional analyses could be completed to add to our understanding of genotype–phenotype relationships in OCNDS.

## Data Availability

The datasets presented in this study can be found in online repositories. The names of the repository/repositories and accession number(s) can be found by applying at: https://base.sfari.org/, SFARI Base.

## Footnotes

^1^ https://www.simonssearchlight.org/

^2^ https://gene.sfari.org/about-sfari-gene/

^3^ https://www.pearsonassessments.com/en-us/Store/Professional-Assessments/Behavior/Vineland-Adaptive-Behavior-Scales-%7C-Third-Edition/p/100001622

^4^ https://docs.google.com/viewer?url=https%3A%2F%2Fcdn.simonssearchlight.org%2Fwp-content%2Fuploads%2F2022%2F02%2F23033256%2FCSNK2A1_OCNDS_VoC.pdf

^5^ https://docs.google.com/viewer?url=https%3A%2F%2Fcdnsimonssearchlight.org%2Fwp-content%2Fuploads%2F2022%2F02%2F23033256%2FCSNK2A1_OCNDS_VoC.pdf

^6^ https://www.simonssearchlight.org/
