# Supplementary material for: OCNDS core features are conserved across variants, with loop-region mutations driving greater symptom burden
Source: Front Hum Neurosci. 2025 Jul 3;19:1589897. doi: 10.3389/fnhum.2025.1589897 (PMC12267189; doi:10.3389/fnhum.2025.1589897)
Supplement: Supplementary file 1 [file Supplementary_file_1.docx]

**Supplemental Figure Legends**

**Supplemental Figure 1.** Symptoms reported in OCNDS patients harboring missense variants plotted alongside nonsense, deletion, frameshift, splice, and duplication variants. Comparison via Kruskal-Wallis test was significant (p=0.02) however, subsequent Dunn’s multiple comparisons test did not yield significant differences between groups.

**Supplemental Figure 2.** Comparisons of the number of symptoms across symptom domains between loop and non-loop *CSNK2A1* patients. All comparisons were not statistically significantly different between groups (Mann Whitney tests). (A) allergy (e.g., allergy, medication allergy; p=0.57), (B) autoimmune (e.g., chronic fatigue, Hashimoto’s thyroiditis; p>0.99), (C) cardiac (e.g., congenital heart disease, dilation of aorta; p=0.57), (D) dermatological (e.g., café au lait, hemangioma; p>0.99), (E) Endocrine (e.g., failure to thrive, short stature, hypothyroidism, late puberty, irregular menses; p=0.43), (F) genitourinary (e.g., undescended testicles, bicornate uterus; p=0.17), (G) infectious diseases (e.g., otitis media, pneumonia, urinary tract infection; p=0.95), (H) orthopedic (e.g., kyphosis, scoliosis, vertebral anomalies, hip dysplasia, pectus carinatum, pectus excavatum; p>0.99), (I) surgeries (e.g., cancer, cardiac, gastronomy tube, craniofacial – cleft repair; p=0.89), (J) visual (e.g., astigmatism, coloboma, crossed eyes, farsighted, lazy eye, ptosis, nearsighted, depth perception issue, repetitive eye movements; p=0.11), (K) gastrointestinal (e.g., celiac, diarrhea, constipation, GERD) (p=0.09), (L) joint (e.g., hypermobile joints) (p>0.99), and (M) neurological seizure symptoms (e.g., atonic drop attack, grand mal, petit mal, complex partial, infantile spasms) (p=0.86). (N) The number of reported medications was not different between groups (Mann Whitney test, p=0.29). Specific symptom subdomains were compared, and no significant differences were found between loop and non-loop patients (Fisher’s exact tests) when comparing (O) GERD (p=0.32), (P) astigmatism (p=0.06), (Q) failure to thrive (p=0.45), (R) short stature (p=0.26), and (S) microcephaly (p=0.41).

**Supplemental Figure 3.** Comparing developmental milestones between loop and non-loop patients. All comparisons were not statistically significantly different (Mann Whitney Tests). (A) Age when bowel trained (p=0.69); 7 individuals with loop variants (ages: 1.4, 1.8, 3.8, 4.6, 5.2, 8.5, 11.8) and 1 with a non-loop variant (age 3.6) reported ‘not yet’ (*data not shown,* S2A). (B) Age when bladder trained (p=0.47); 5 individuals with loop variants (ages: 1.4, 1.8, 3.8, 4.6, 8.5) and 1 individual with a non-loop (age 3.6) variant reported ‘not yet’ and 1 individual with a loop variant reported bladder control after 7 years of age (*data not shown*) S2B). (C) Age when first crawled (p=0.11) (S2C). (D) Age when first walked (p=0.86); 2 individuals with loop variants reported ‘not yet’ (ages 1.4 and 1.8, data not shown, S2D). The outlier at 240 months has an E180K mutation in the activation loop. (E) Age when first sat up without support (p=0.20). (F) Age at seizure onset was reported for 11 individuals; statistical analysis could not be completed due to only one case reported for non-loop variants.

**Supplemental Figure 4.** Percentage of OCNDS patients with specified symptoms. Red reflects total OCNDS missense cohort, teal reflects individuals with loop missense variants, black reflects individuals with non-loop missense variants, and orange represents individuals reported in the literature. Note: Symptoms in orange may differ from our study due to limitations in published case reports, including inconsistent terminology and reporting standards.

**Supplemental Figure 5.** Subset of domain scores from Vineland-III survey are expressed as standard scores with a mean of 100 and standard deviation of 15. Subdomain scores are plotted as raw scores. All comparisons were not statistically significantly different between loop (n=25) and non-loop (5) individuals (Mann Whitney Tests). (A) Adaptive Behavior Composite (ABC) Score (p=0.83). (B) Daily Living Skills Domain (p =0.88). (C) Socialization Skills Domain (p =0.73). (D) Communication Skills Domain (p=0.82). (E) Motor Skills Domain (p =0.31). For subdomains, 24 loop and 5 non-loop patients were compared; (E’) Fine Motor Skills Subdomain (p =0.19) and Gross Motor Skills Subdomain (p =0.39).

**Supplemental Figure 6.** Comparisons of the number of symptoms across symptom domains between individuals with CK2β-binding and non-binding variants. All comparisons were not significantly different via Mann Whitney tests. (A) Endocrinology (p=0.92). (B) Dermatological (p>0.99). (C) Genitourinary (p=0.39). (D) Gastrointestinal (p=0.79). (E) Seizure (p=0.93) (F) Surgeries (p=.63). (G) Visual (p=0.07).

**Supplemental Table 1: Patients Excluded from Analysis**

^*^Individuals have additional 'pathogenic/ likely pathogenic' mutations in other genes that may contribute to disease phenotype. VUS = variants of unknown significance.

**Supplemental Table 2: Individual-level patient symptom burden.**

Numerical identifier is arbitrary and does not represent any identifier utilized in Simons Searchlight; thus, individual-level reporting of symptom burden is de-identified. Green colored box indicates presence of symptom and gray colored box indicates absence of symptom. *in SD/SD indicates individual reported to be non-verbal after 48 months. * in Sleep indicates individual meets the clinical cutoff for sleep disorders utilizing the CSHQ. Abbreviations: SD/SD = Speech Disorders/Speech Delay, Neuro = Neurological Non-Seizure, GDD= Global Developmental Delay, ID/LD = Intellectual Disability/Learning Disability, GI = Gastrointestinal Disorders, Endo = Endocrine Disorders, Inf Dis = Infectious Diseases, ASD=Autism Spectrum Disorder, Derm = Dermatological, GU = Genitourinary, Resp = Respiratory, Ortho = Orthopedic, Imm Def = Immunodeficiency, AI = Autoimmune Disorders, SBD = Structural Birth Defects.

**Supplemental Table 3. Patient-level symptom burden as reported in the literature.**

Green colored box indicates presence of symptom and gray colored box indicates absence of symptom. ‘NR’ indicates that symptom was not reported (unable to determine based on publication description). *Indicates individuals are related. + indicates individuals are half-siblings. Colored boxes in ‘Sex’ column indicate distinct families. Abbreviations: SD/SD = Speech Disorders/Speech Delay, Neuro = Neurological Non-Seizure, GDD= Global Developmental Delay, ID/LD = Intellectual Disability/Learning Disability, GI = Gastrointestinal Disorders, Endo = Endocrine Disorders, Inf Dis = Infectious Diseases, ASD=Autism Spectrum Disorder, Derm = Dermatological, GU = Genitourinary, Resp = Respiratory, Ortho = Orthopedic, Imm Def = Immunodeficiency, AI = Autoimmune Disorders, SBD = Structural Birth Defects.
