# Supplementary material for: OCNDS core features are conserved across variants, with loop-region mutations driving greater symptom burden
Source: Front Hum Neurosci. 2025 Jul 3;19:1589897. doi: 10.3389/fnhum.2025.1589897 (PMC12267189; doi:10.3389/fnhum.2025.1589897)
Supplement: Supplementary file 2 [file Table_1.docx]

**Supplemental Table 1: Patients Excluded from Analysis**

^*^Individuals have additional 'pathogenic/ likely pathogenic' mutations in other genes that may contribute to disease phenotype. VUS = variants of unknown significance.

| Mutation Type | Number of Patients |
| --- | --- |
| Nonsense | 1 |
| Frameshift | 1 |
| Splice | 2 |
| Duplication | 1 |
| Deletions | 3 |
| VUS | 6 |
| Confounding Variants | 10 |
