# Supplementary figures and images for: OCNDS core features are conserved across variants, with loop-region mutations driving greater symptom burden
Source: Front Hum Neurosci. 2025 Jul 3;19:1589897. doi: 10.3389/fnhum.2025.1589897 (PMC12267189; doi:10.3389/fnhum.2025.1589897)

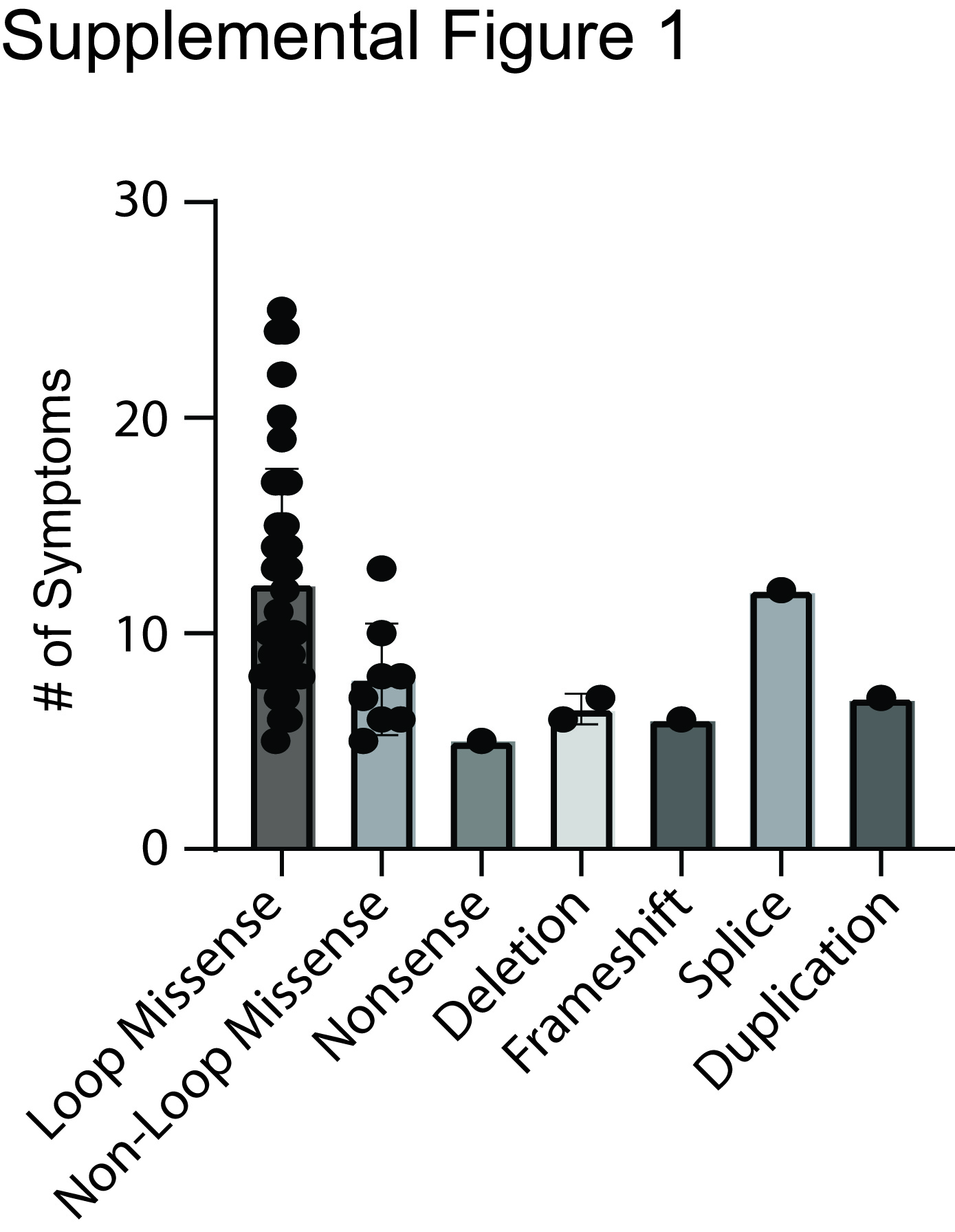

Supplement: Supplementary file 5 [file Image_1.jpg]

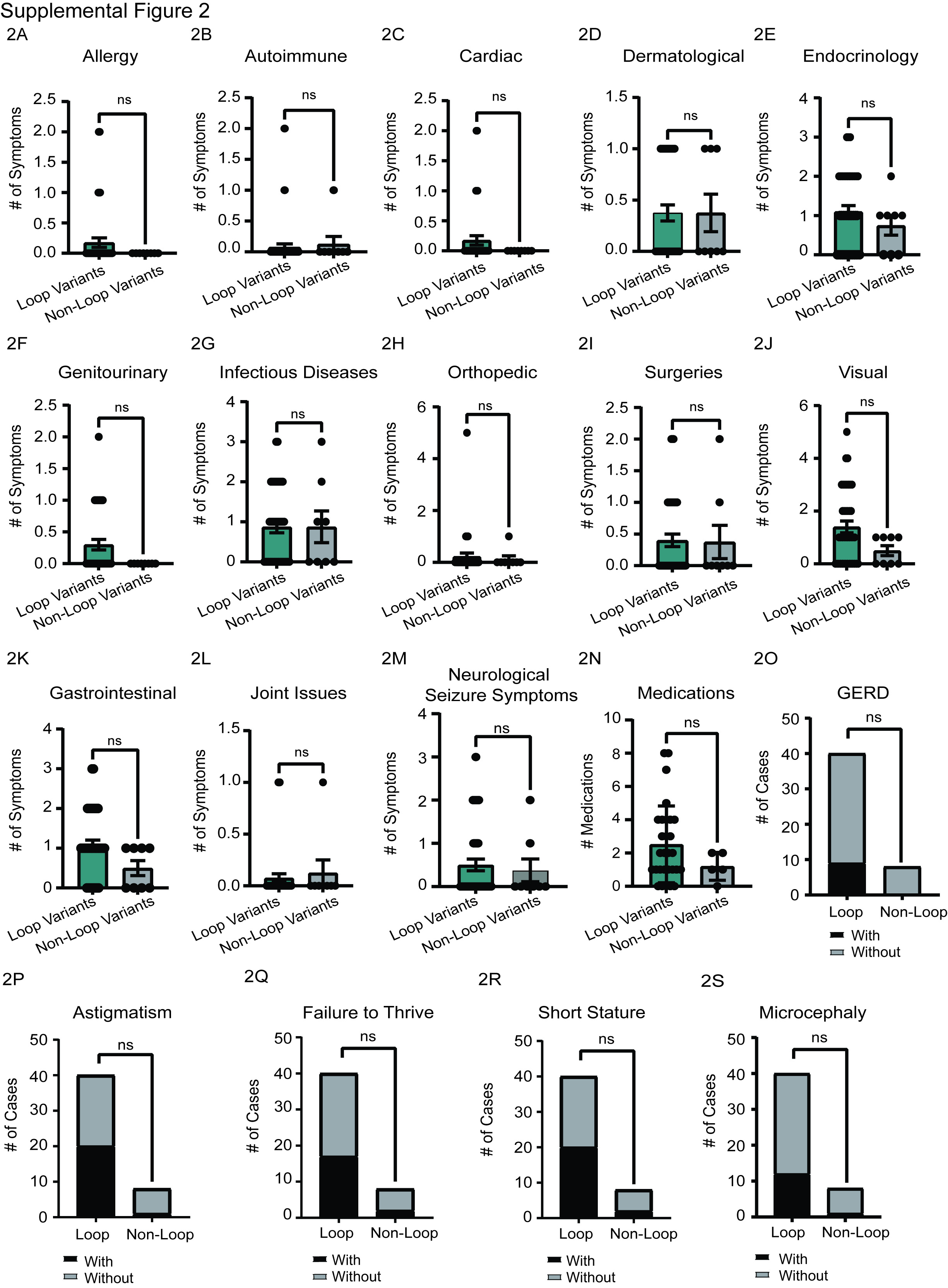

Supplement: Supplementary file 6 [file Image_2.jpg]

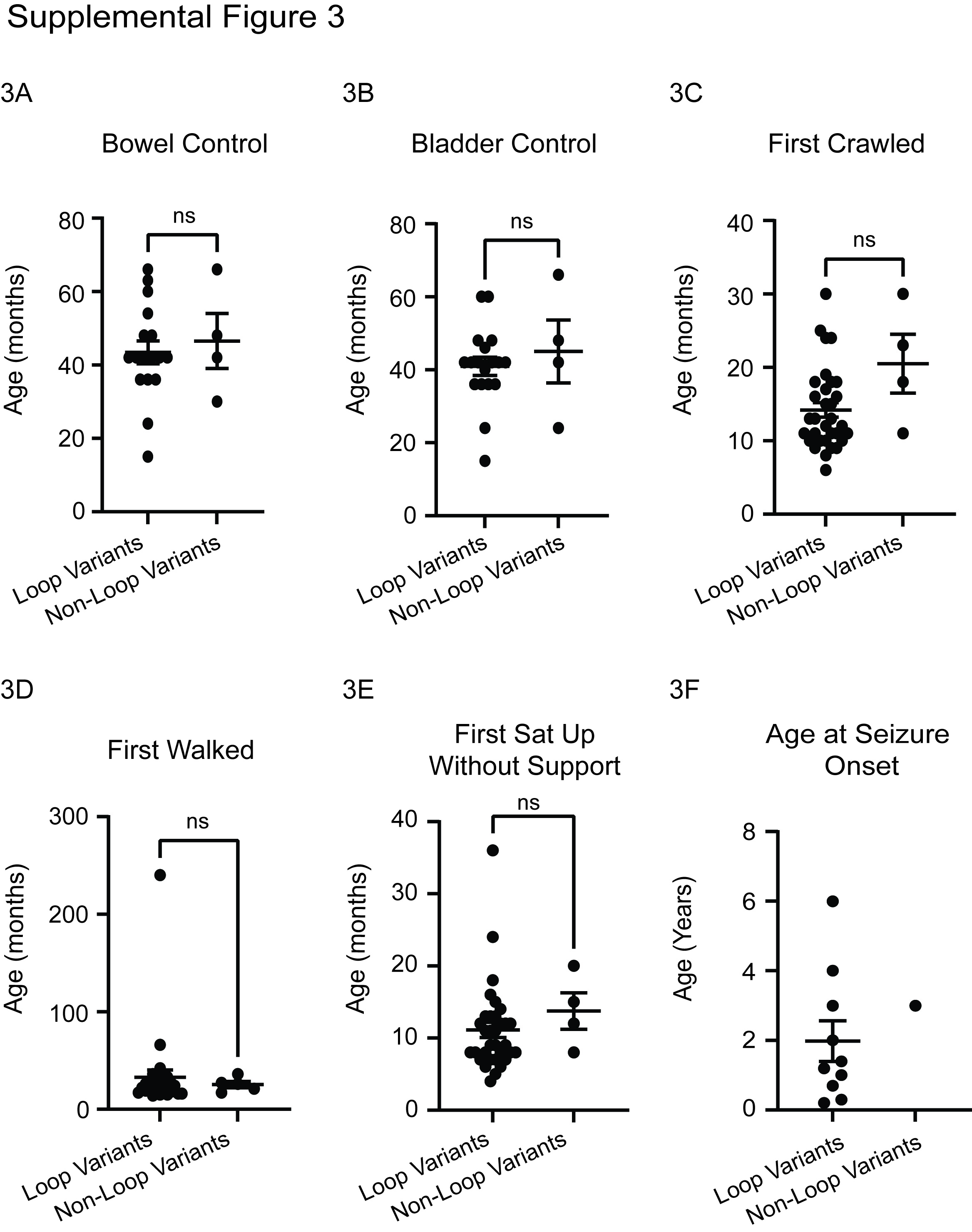

Supplement: Supplementary file 7 [file Image_3.jpg]

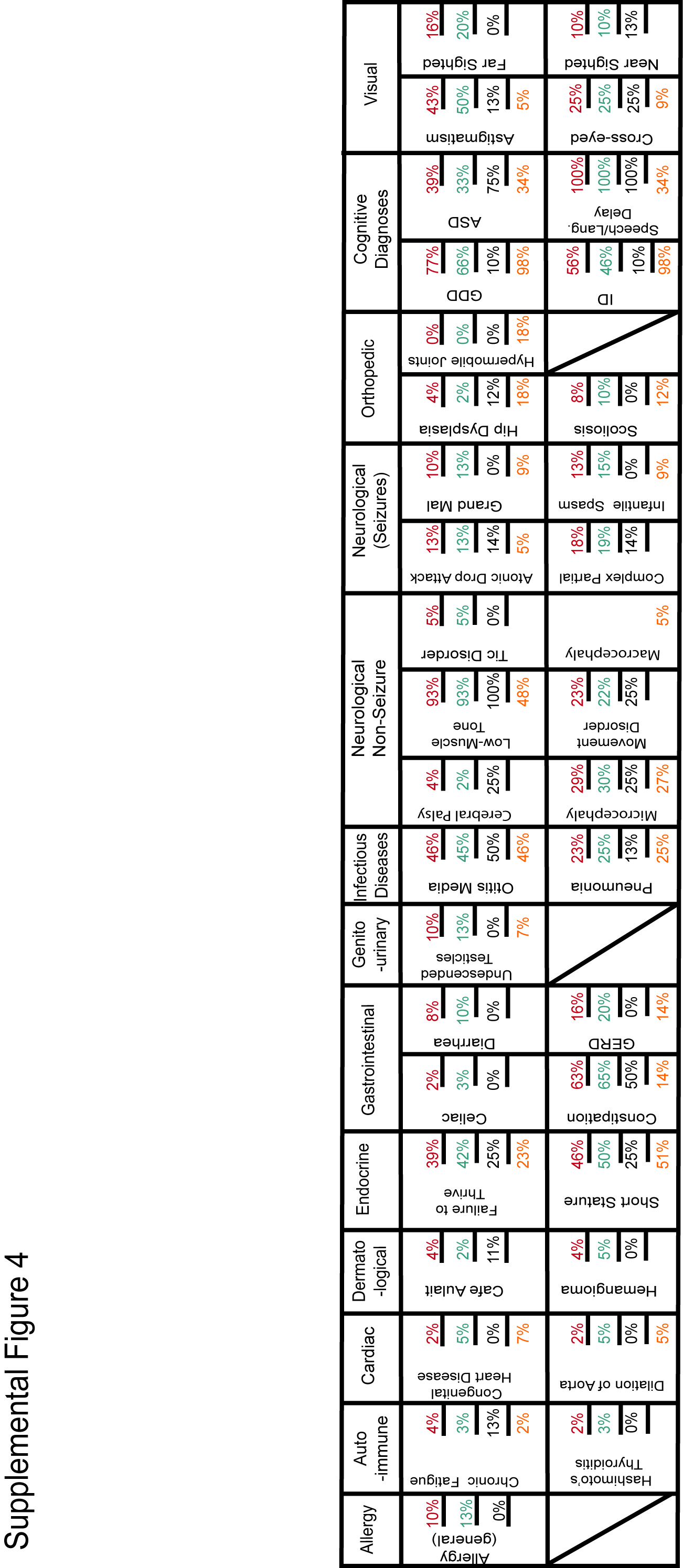

Supplement: Supplementary file 8 [file Image_4.jpg]

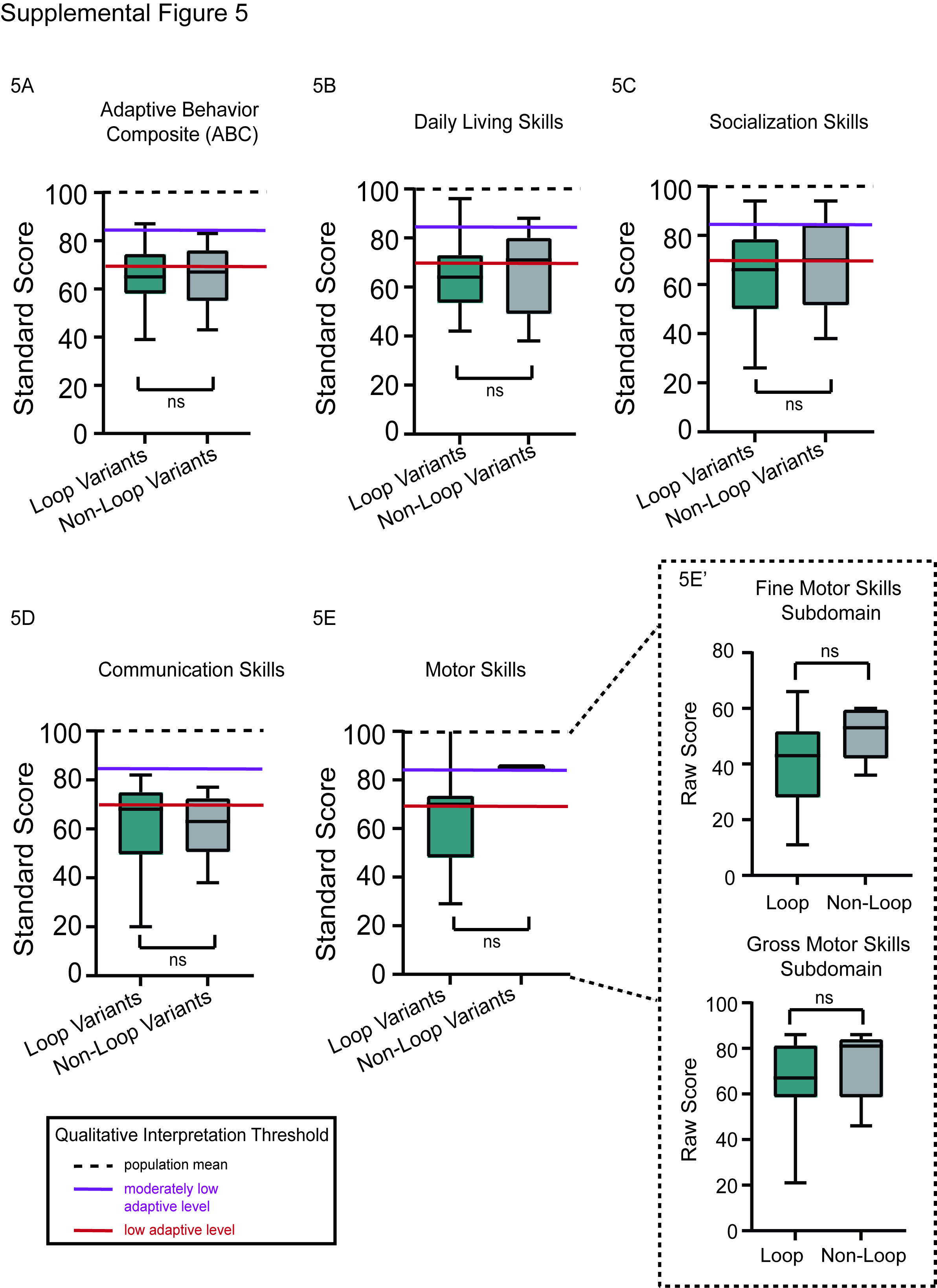

Supplement: Supplementary file 9 [file Image_5.jpg]

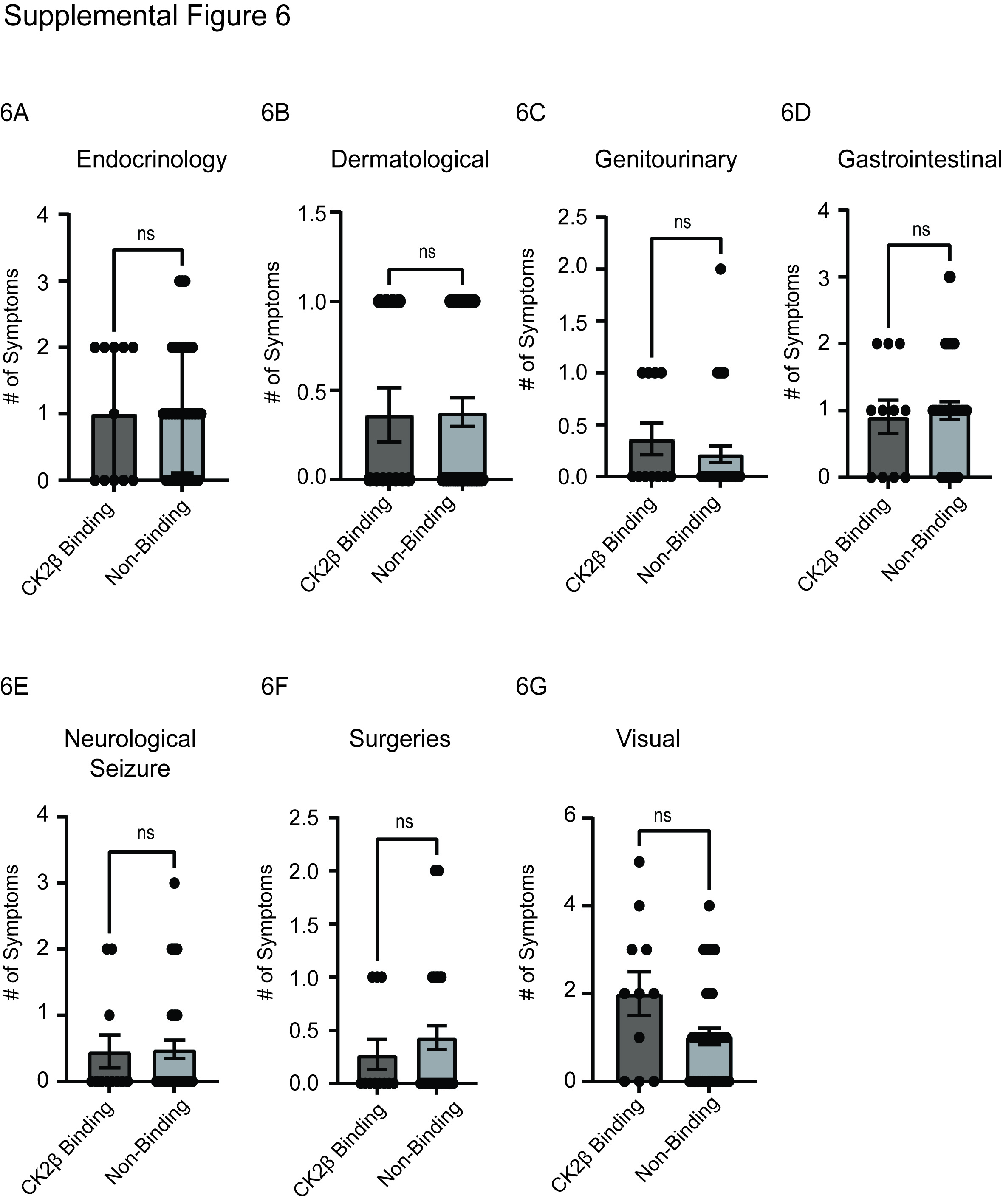

Supplement: Supplementary file 10 [file Image_6.jpg]
